# Explaining the decline in coronary heart disease mortality in Turkey between 1995 and 2008

**DOI:** 10.1186/1471-2458-13-1135

**Published:** 2013-12-05

**Authors:** Belgin Unal, Kaan Sözmen, Hale Arık, Gül Gerçeklioğlu, Deniz Utku Altun, Hatice Şimşek, Sinem Doganay, Yücel Demiral, Özgür Aslan, Kathleen Bennett, Martin O´Flaherty, Simon Capewell, Julia Critchley

**Affiliations:** 1 Department of Public Health, Faculty of Medicine, Dokuz Eylul University, Izmir, Turkey; 2 Ordu Community Health Centre, Ordu, Turkey; 3 Department of Cardiology, Faculty of Medicine, Dokuz Eylul University, Izmir, Turkey; 4 Department of Pharmacology & Therapeutics, Trinity College, Dublin, Ireland; 5 Department of Public Health, University of Liverpool, Liverpool, UK; 6 Division of Population Health Sciences and Education, St George’s, University of London, London, UK

**Keywords:** Coronary heart disease, Coronary heart disease mortality, Coronary heart disease risk factors, Coronary heart disease management, Turkey, Modelling

## Abstract

**Background:**

Coronary heart disease (CHD) mortality rates have been decreasing in Turkey since the early 1990s. Our study aimed to determine how much of the CHD mortality decrease in Turkey between 1995 and 2008 could be attributed to temporal trends in major risk factors and how much to advances in medical and surgical treatments.

**Methods:**

The validated IMPACT CHD mortality model was used to combine and analyse data on uptake and effectiveness of CHD treatments and risk factor trends in Turkey in adults aged 35–84 years between 1995 and 2008.

Data sources were identified, searched and appraised on population, mortality and major CHD risk factors for adults those aged 35–84 years. Official statistics, electronic databases, national registers, surveys and published trials were screened from 1995 onwards.

**Results:**

Between 1995 and 2008, coronary heart disease mortality rates in Turkey decreased by 34% in men and 28% in women 35 years and over. This resulted in 35,720 fewer deaths in 2008.

Approximately 47% of this mortality decrease was attributed to treatments in individuals (including approximately 16% to secondary prevention, 3% angina treatments, 9% to heart failure treatments, 5% to initial treatments of acute myocardial infarction, and 5% to hypertension treatments) and approximately 42% was attributable to population risk factor reductions (notably blood pressure 29%; smoking 27%; and cholesterol 1%). Adverse trends were seen for obesity and diabetes (potentially increasing mortality by approximately 11% and 14% respectively). The model explained almost 90% of the mortality fall.

**Conclusion:**

Reduction in major cardiovascular risk factors explained approximately 42% and improvements in medical and surgical treatments explained some 47% of the CHD mortality fall. These findings emphasize the complimentary value of primary prevention and evidence-based medical treatments in controlling coronary heart disease.

## Background

Turkey has a relatively young population compared to many western societies. The total population was 75 million in 2008 and 30% were younger than 15 years of age [1]. However, population structure is changing rapidly and Turkey has almost completed its demographic transition [2]. The median age of the population thus increased from 22 years in 1990 to 27 years by 2008. The proportion of those over 65 years of age was approximately 4% in 1990 and reached to 7% in 2008 [2]. The ageing population and changes in lifestyle have contributed to increases in the burden of non-communicable diseases (NCDs) [3].

Coronary heart disease (CHD) is the major cause of death and disability in Turkey. In the Turkish National Burden of Disease Study (2000) CHD and cerebrovascular diseases were estimated to account for 36% of all deaths [3].

In most industrialized countries, CHD mortality rates have decreased considerably since the 1970s [4]. Decreases started some 10–15 years later in middle income countries [4]. Thus in a recent analysis of CHD mortality rates in Turkey, increasing trends were observed from 1988 (366 per 100000) to 1994 (411 per 100000) but then decreasing from 1995 to reach (348 per 100000 in 2008) [5]. However, the underlying factors associated with this significant decrease in CHD mortality have not been comprehensively evaluated.

Previous studies in some high income countries have shown that improvements in major cardiovascular risk factors, including total cholesterol, blood pressure levels and smoking, explain a greater proportion of the mortality decline than treatments, ranging from 44% in the USA to 72% in Finland [6-8]. This principally reflects improvements in smoking and modifiable dietary risk factors [6-8]. However few such studies have been performed in middle or low income countries.

The information on CHD prevalence, incidence and determinants of CHD in Turkey is rapidly improving. There are several ongoing studies from the Turkish Cardiology Association [9], the Turkish Society of Hypertension and Renal Disease [10,11], the Turkish Endocrinology Association (TURDEP) [12,13] and from the Universities [14-17]. These together provide increasing good trend data on cardiovascular risk factors.

Thus, in Turkey, recent population studies document significant decreases in population blood pressure, cholesterol [18,19] and smoking prevalence [20]. However, obesity and diabetes prevalence are now increasing steeply [13,21,22] as in most other developed and developing countries.

Approximately 25%-55% of the recent CHD mortality declines in developed countries have been attributed to the use of evidence-based medical and interventional therapies [7,8,23,24]. These include aspirin, beta-blockers, ACE-inhibitors and angiotensin-receptor-blockers (ARBs), statins, fibrinolysis, percutaneous coronary intervention (PCI) and coronary artery bypass surgery (CABG) [7,25]. In Turkey, treatment uptake in CHD patients has increased substantially since the 1990s [26,27].

Our objective was therefore to study recent trends in CHD deaths between 1995 and 2008, using a previously validated epidemiological model, to determine the contribution of prevention and treatment strategies.

## Methods

We used an updated version of the IMPACT CHD mortality model that was originally developed in Scotland and then validated in many countries including England, New Zealand, United States, Canada, Europe and China [7,24,28-30]. Data on risk factor levels and current uptake levels of evidence based medical and surgical treatments were identified by extensive search of published and unpublished data and complemented with specifically designed surveys. All data sources were critically appraised by the research team and the results are presented in Additional file 1: Table S1.

The IMPACT model was populated with data for men and women aged 35–84 (in 10 year age groups) for both 1995 and 2008. The main data items included data on: a) population sizes in each year, by age group and gender; b) Patient numbers in specific CHD groups (Myocardial Infarction, Congestive Heart Failure, Chronic Angina Pectoris, c) uptake levels of specific medical and surgical treatments, (Table 1), and d) population trends in major cardiovascular risk factors (smoking, total cholesterol, systolic blood pressure, body mass index, diabetes, physical inactivity and fruit and vegetable consumption) (Table 2). The number of CHD deaths attributable to each specific treatment and risk factor were calculated for 1995 and for 2008. The difference between the two values then represented the deaths prevented or postponed (DPP) attributed to the change in risk factors and treatment uptake levels in the population.

**Table 1:** Estimated deaths prevented or postponed by medical or surgical treatments in Turkey in 2008

						**Number of deaths prevented or postponed**			**Percentage of total reduction**		
**Treatments**	**Number of eligible patients**	**Patients receiving treatment (%)**	**Relative risk reduction (%)**	**Mean case-fatality (%)**	**Absolute risk reduction**	**Best estimate**	**Minimum estimate**	**Maximum estimate**	**Best estimate**	**Minimum estimate**	**Maximum estimate**
**Acute MI**	**91317**			**0.067**		**1677**	**684**	**3309**	**4.7**	**1.9**	**9.3**
Community CPR	9132	100	0.05	0.067	0.055	443	283	638	1.2	0.8	1.8
Hospital CPR	2740	6	0.32	0.067	0.323	53	34	91	0.1	0.1	0.3
Thrombolysis	91317	29	0.26	0.067	0.014	316	130	656	0.9	0.4	1.8
Aspirin	91317	93	0.15	0.067	0.010	774	317	1420	2.2	0.9	4.0
Beta blocker	91317	70	0.04	0.067	0.003	143	59	296	0.4	0.2	0.8
ACE inhibitor	91317	61	0.07	0.067	0.005	228	93	466	0.6	0.3	1.3
Primary PTCA	91317	18	0.32	0.067	0.020	589	241	1221	1.6	0.7	3.4
Primary CABG	91317	9	0.20	0.067	0.013	112	40	231			
MI treatments in 1995 subtracted						-1244	-599	-2367	3.5	1.7	6.6
**Unstable angina**						**639**	**364**	**1800**	**1.8**	**1.0**	**5.0**
Aspirin & heparin	45658	50	0.33	0.053	0.017	316	129	655	0.9	0.4	1.8
Aspirin alone	45658	88	0.15	0.053	0.008	245	100	465	0.7	0.3	1.3
PG IIB/IIIA	45658	41	0.09	0.053	0.005	70	28	144	0.2	0.1	0.4
CABG surgery for UA	45658	13	0.43	0.053	0.023	107	44	221	0.3	0.1	0.6
PTCA for UA	45658	26	0.32	0.053	0.016	152	62	314	0.4	0.2	0.9
**Secondary Prev Post AMI**	**759071**			**0.061**		**4548**	**1454**	**6509**	**12.7**	**4.1**	**18.2**
Aspirin	759071	73	0.778	0.045	0.007	2170	711	3665	6.1	2.0	10.3
Beta blocker	759071	63	0.526	0.045	0.010	2052	672	3713	5.7	1.9	10.4
ACE inhibitor	759071	61	0.514	0.045	0.009	1771	580	3198	5.0	1.6	9.0
Statin	759071	3	0.757	0.045	0.010	1339	351	2767	3.7	1.0	7.7
Warfarin	759071	8	0.032	0.045	0.010	76	25	138	0.2	0.1	0.4
**Secondary Prev Post CABG/PCI**	**275652**			**0.006**		**1752**	**575**	**4164**	**4.9**	**1.6**	**11.7**
Aspirin	273379	97	0.15	0.017	0.003	496	162	1076	1.4	0.5	3.0
Beta blocker	273379	60	0.23	0.017	0.004	470	154	1167	1.3	0.4	3.3
ACE inhibitor	273379	54	0.20	0.017	0.003	366	120	909	1.0	0.3	2.5
Statin	273379	87	0.22	0.017	0.004	377	124	898	1.1	0.3	2.5
Warfarin	273379	2.2	0.22	0.017	0.004	18	6	44	0.1	0.0	0.1
Rehabilitation	273379					83	27	208	0.2	0.1	0.6
**Chronic angina**						**3348**	**1105**	**8627**	**9.4**	**3.1**	**24.2**
CABG surgery 1995-2008	103293	100	0.35	0.012	0.002	632	324	1093	1.8	0.9	3.1
CABG treatments in 1995 subtracted	103293					-6	-4	-8	0.0	0.0	-0.1
Angioplasty 1996-2000	103293	100	0.00	0.022	0.000	0	0	0	0.0	0.0	0.0
Aspirin in community	103293	78	0.15	0.012	0.001	2840	931	7066	8.0	2.6	19.8
Statins in community	103293	63	0.23	0.012	0.003	1517	398	4530	4.2	1.1	12.7
**Hospital heart failure**	**46511**			**0.258**		**1025**	**306**	**2756**	**2.9**	**0.9**	**7.7**
ACE inhibitor	46511	43	0.20	0.166	0.033	251	66	744	0.7	0.2	2.1
Beta blocker	46511	37	0.35	0.166	0.058	378	99	1121	1.1	0.3	3.1
Spironolactone	46511	40	0.30	0.166	0.051	510	167	1269	1.4	0.5	3.6
Aspirin	46511	68	0.15	0.166	0.025	428	140	1041	1.2	0.4	2.9
**Community heart failure**	**424611**			**0.085**		**2338**	**747**	**5961**	**6.5**	**2.1**	**16.7**
ACE inhibitor		70	0.20	0.085	0.013	479	126	1430	1.3	0.4	4.0
Beta blocker		70	0.35	0.085	0.030	986	323	2453	2.8	0.9	6.9
Spironolactone		25	0.31	0.085	0.030	883	289	2197	2.5	0.8	6.1
Aspirin		90	0.15	0.085	0.013	924	303	2299	2.6	0.8	6.4
Statins		40	0.00	0.085	0.000	0	0	0	0.0	0.0	0.0
**Hypertension treatment**	**10029567**	**57**	**0.13**	**0.007**	**0.001**	**2153**	**356**	**4472**	**6.0**	**1.0**	**12.5**
**Statins for primary prevention**	**6133187**	**2**	**0.35**	**0.006**	**0.002**	**41**	**11**	**205**	**0.1**	**0.0**	**0.6**
											
**Total treatment**						**16671**	**5356**	**35362**	**47.0**	**15.1**	**100.0**

**Table 2:** Deaths from coronary heart disease prevented or postponed as a result of changes in population risk factors in Turkey, 1995-2008

**Risk factor**	**Number of eligible patients**	**Absolute level of risk factors**		**Change in risk factors**		**Beta regression coefficient for change in mortality rate**	**Relative risk**	**Deaths prevented or posponed**					
		**1995**	**2008**	**Absolute change**	**Relative change (%)**			**Number of deaths**			**Percent of total reduction**		
								**Best estimate**	**Minimum estimate**	**Maximum estimate**	**Best estimate**	**Minimum estimate**	**Maximum estimate**
Smoking prevalence, %	27186380	27.0	16.0	-11.0	-36.1			9724	7779	11669	27.2	21.8	32.7
Men	13497080	44.4	24.6	-19.8	-45.5		2.52	8377	6702	10053	48.8	39.0	58.6
Women	13689300	9.4	7.2	-2.2	-27.2		2.14	1347	1077	1616	7.3	5.8	8.7
Systolic BP, mmHg	27186380	127.3	124.6	-2.8	-2.0			13549	8889	18937	37.9	24.9	53.0
Men	13497080	124.1	123.3	-0.7	-0.0	-0.033		3579	2318	5081	15.8	10.3	22.5
Women	13689300	130.2	125.8	-4.4	-3.5	-0.041		9970	6572	13855	55.9	36.8	77.6
Population BP after adjustment for hypertension treatments								10363	8263	12990	29.0	23.1	36.4
Total cholesterol, mmol/L	27186380	4.96	4.97	0.01	0.01			-354	-226	-510	-1.0	-0.6	-1.4
Men	13497080	4.91	4.92	0.01	0.01	-0.627		-200	-128	-288	-0.0	0.0	0.1
Women	13689300	5.02	5.03	0.01	0.01	-0.619		-154	-99	-222	-0.0	0.0	0.1
Population TC after adjustment for antilipidemic treatments								-395	-237	-715	1.1	0.7	2.0
BMI, kg/m^2^	27186380	27.4	29.0	1.59	6.0			-3790	-2149	-5784	10.6	6.0	16.2
Men	13497080	26.1	27.9	1.80	7.0	0.028		-2117	-1202	-3227	12.3	7.0	18.8
Women	13689300	28.7	30.1	1.37	5.0	0.028		-1673	-947	-2557	9.0	5.1	13.8
Diabetes prevalence, %	27186380	15.5	18.7	3.2	18.7			-5086	-4069	-6103	-14.2	11.4	17.1
Men	13497080	14.5	17.5	3.0	20.4		1.93	-1866	-1493	-2240	-10.9	8.7	13.0
Women	13689300	16.4	19.9	3.4	17.2		2.59	-3220	-2576	-3864	-17.4	13.9	20.8
Physical inactivity	27186380	70.0	65.0	-5.0	-7.0			1919	1535	2303	5.4	4.3	6.4
Mean Fruit&Vegetable consumption in portions	27186380	3.2	3.5	32.0	10.0	0.041		2377	475	4974	6.7	1.3	13.9
Total risk factors								15112	11597	19333	42.3	32.5	57.3

BMI = weight [kg]/height [m]^2^.

Information on population and demographic changes were obtained from the census (1995) and Address Based Population Registration System (2008) of the Turkish Statistical Institute [1]. Although the data collection method changed from 1995 to 2008, population projections based on the census are very close to the population of address based registration in 2008 [31].

### The numbers of CHD deaths were estimated using mortality statistics from the Turkish statistical institute

Data on cause of death was collected only from urban settings which comprised approximately 50%-68% of the total population over the years 1988–2008 [1]. Therefore, the total number of deaths was estimated by inflating the urban deaths proportional to the rural population simply assuming that a similar mortality pattern existed in rural areas [3]. The total number of deaths was also inflated by 12% in men and 16% in women to account for underreporting of the deaths based on expert opinion [3]. The numbers of CHD deaths by age and sex groups for 1995 and 2008 were obtained from the Turkish Statistical Institute coded according to International Classification of Diseases, 8^th^*Revision* (corresponds to ICD9 codes 410–414) based on final cause of death [1]. Coding inaccuracy is an important limitation of the mortality data in Turkey [32]. Ill defined codes and the “other heart diseases” codes accounted for approximately 30 to 40% of the total deaths during the study period [32]. Therefore, 50% of the “other heart disease” group were allocated to CHD deaths through the study period [3] .

### Population risk factor trend data

For the base year of 1995 were obtained from national representative surveys TEKHARF [9], TURDEP [12] supplemented with regional studies [15,33-35]. For the later years more national surveys became available [15-17]. The main data sources for major risk factors are presented in Additional file 1: Table S2.

### The numbers of hospital admissions for CHD

Were estimated using the Ministry of Health (MoH) hospital admission data [36] and from two cohort studies [37,38]. The number of patients undergoing Coronary Artery Bypass Grafting (CABG) and angioplasty were obtained from the MoH hospital admission data supplemented with information by experts from the Turkish Cardiology Association and Cardiovascular Surgery Association, emergency physicians and family physicians. In total 10 experts were initially contacted individually and sent a questionnaire asking their opinions on number of hospital admissions and treatment uptakes for CHD patient groups. Following this they were invited to participate a consensus meeting with other experts. Treatment uptake levels for initial CHD treatments in the hospital were obtained from the hospital survey designed by the research team in the Dokuz Eylul University Hospital and studies published on treatment uptake in the emergency department [39].

The prevalence of angina, post myocardial infarction patients and congestive heart failure in the community was estimated based on representative population surveys including the National Household Survey [3] and Health Surveys 2008 and 2010 [40,41].

Information on treatment uptake in the community was based on PREMISE [27], EUROASPIRE III [26] and population based studies [14,42,43].

### The efficacy of specific therapeutic interventions

Was based on recent meta-analyses and randomised controlled trials (Additional file 1: Table S4). The potential effect of multiple treatments in an individual patient was quantified using the Mant and Hicks **cumulative relative benefit** approach [44] as detailed in Additional file 1.

#### Change in CHD Deaths between 1995 and 2008

The number of CHD deaths expected in 2008 assuming that mortality rates in 1995 persisted to 2008 was calculated by indirect age standardization. The CHD deaths actually observed in 2008 were then subtracted to quantify the decrease in CHD deaths between 1995 and 2008. These represent the number of deaths prevented or postponed (DPPs) between 1995 and 2008 which the model needs to explain.

#### The mortality changes attributed to risk factor trends

The DPPs from changes in risk factors were estimated using two approaches: ***The regression β coefficient approach*** was used to quantify the population mortality impact of change in those specific risk factors, measured as continuous variables, (systolic blood pressure, total cholesterol and BMI). The mortality reduction was then estimated as the product of the CHD deaths observed in 1995 (the baseline year), the subsequent reduction in that risk factor and the regression coefficient that quantified the change in CHD mortality expected per unit of absolute change in the risk factor. The sources of regression were presented in Additional file 1: Table S5.

The second approach, ***population attributable risk fraction***(PAR) was used for categorical variables- smoking, diabetes, physical inactivity and fruit and vegetable consumption using Levin’s equation:

$$\text{PAR}=\frac{\text{Prevalence}\times \left(\text{Relative}\;\text{Risk}-1\right)}{\left[\text{Prevalence}\times \left(\text{Relative}\;\text{Risk}-1\right)+1\right]}$$

Details of the model methodology have been published previously [7,33] and are illustrated in Additional file 1.

#### Estimating the contribution of medical and surgical treatments

The model included all medical and surgical treatments in 1995 (the base year) and 2008 (the final year). Treatment uptake data was limited for the year 1995 and thus much of the data included in the model for this year was estimated based on opinion of cardiology experts from Dokuz Eylul University (Additional file 1: Table S2).

The treatment arm of the model consisted of mutually exclusive CHD subgroups: patients hospitalized within the last year for an acute myocardial infarction (AMI), or for unstable angina pectoris or heart failure due to ischemic cardiomyopathy, community-dwelling patients who were post-AMI survivors, patients with chronic angina, patients receiving revascularisation CABG surgery or angioplasty, patients with heart failure in the community and, finally, hypertensive and hypercholesterolemia individuals eligible for primary prevention with lipid lowering therapy.

The mortality reduction for each treatment within each patient group was then calculated as the product of:

The number of patients in that group, their age-specific case fatality, the patient uptake (the proportion receiving that specific treatment) and treatment efficacy (the relative mortality reduction reported in published meta-analyses and trials) Additional file 1.

Case-fatality data were obtained from large, unselected, population-based patient cohorts [45,46]. Survival benefit over a one-year time interval was used for all treatments [47] stratified by age and sex.

#### Treatment adherence and overlaps

Potential overlaps between different groups of patients were identified and appropriate adjustments were made. Patients group calculations and assumptions are detailed in Additional file 1.

Adherence or the proportion of treated patients actually taking therapeutically effective levels of medication, was assumed to be 100% in hospital patients, 70% in symptomatic community patients, and 50% in asymptomatic community patients based on the available literature [48].

#### Sensitivity analyses

Because of the uncertainties surrounding many of the values, a multi-way sensitivity analysis was performed using the analysis of extremes method [49]. For each model parameter, a maximum and minimum feasible value was assigned using the 95% confidence intervals from the source documentation when this was available. We otherwise robustly defined these limits as 20% above and below the best estimate (for the number of patients, use of treatment, compliance and case fatality)*.*

#### Validation: comparison of model estimates with observed mortality falls

The model estimates for the total number of deaths prevented or postponed by each treatment and each risk factor change were summed and compared with the observed changes in mortality for men and women in each age group. Any shortfall in the overall model estimate was then presumed to be attributable either to inaccuracies in our model estimates or to other, unmeasured risk factors [7,24,28]. The study protocol was approved by the Izmir Clinical Research Ethics Commitee (Decision no:09-9/15 Date:07.09.2009)

## Results

Between 1995 and 2008, age-adjusted CHD mortality rates in Turkey fell 34% in men (from 455.9 to 365.7 per 100 000 aged 35–84 years) and 28% in women (from 378.6 to 324.5 among women aged 35–84 years). In 1995, there were 79,065 deaths among this age group due to CHD. In 2008, a total of 96,365 CHD deaths were recorded representing 35,720 fewer CHD deaths than expected if the baseline mortality rates in 1995 had persisted.

Overall, the Turkish IMPACT model was able to explain approximately 31,785 (89%) of the 35715 mortality decrease between 1995 and 2008. The remaining 11% was attributed to changes in other, unmeasured factors.

### Medical and surgical treatments

**Figure 1:**
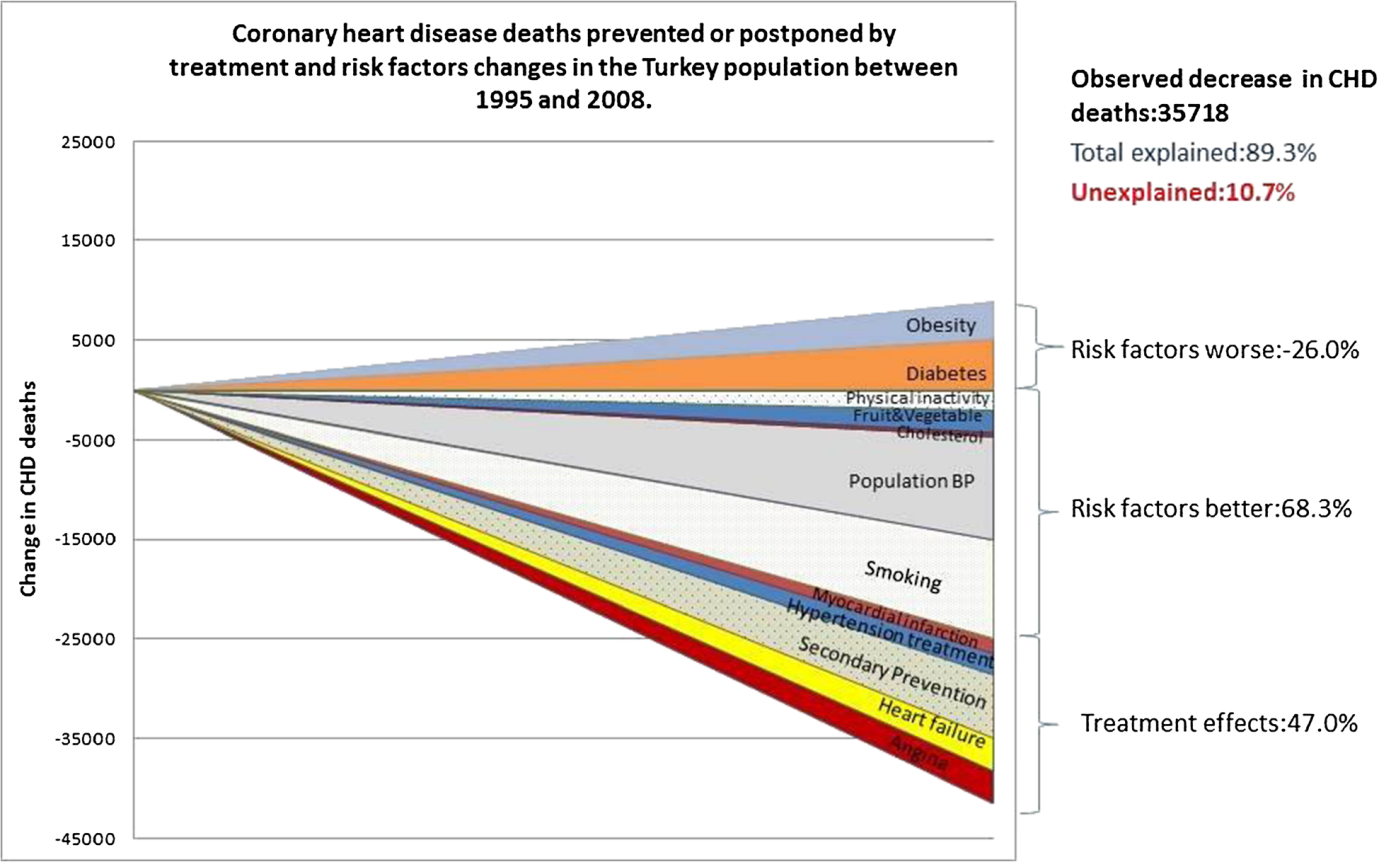
Coronary heart disease deaths prevented or postponed by treatment and risk factors changes in the Turkey population between 1995 and 2008.

Improvements in medical and surgical treatments between 1995 and 2008 prevented or postponed approximately 16,670 CHD deaths (*minimum estimate****5355****, maximum estimate****35360****Table *1*, Figure *1) representing approximately 47% of the overall CHD mortality reduction.

The most substantial contributions came from the improved treatment of post AMI patients (approximately 4550 fewer deaths; 13% of total; m*inimum estimate 1455, maximum estimate 6509)* Aspirin, Beta blockers, ACE inhibitors and Statins each provided similar benefits.

Secondary prevention following revascularisation had a modest effect on CHD mortality reduction with a contribution of 5% *(minimum 1.6% and maximum 11.6%).*

The second largest contribution came from chronic angina treatments (a total reduction of approximately **3350** deaths, representing some 9% of total mortality reduction).

Heart failure treatments in the community and immediate treatments for AMI and unstable angina each explained approximately 6% of the mortality fall. Primary PTCA used in treatment for AMI was associated with a relatively modest reduction in mortality, saving approximately 590 lives and representing approximately 1.6% of the total.

Small mortality reductions were also explained by the treatments for hypertension (5%) and statins in primary prevention (4%) and heart failure treatments in the hospital (3%) (Table 1).

### Major cardiovascular risk factors

Changes in major cardiovascular risk factors together explained approximately 15,110 CHD deaths prevented or postponed *(minimum estimate 11595, maximum estimate 19335) (Table *2*),* representing approximately 42% of the CHD mortality fall between 1995 and 2008. The largest reduction in deaths was explained by substantial reductions in population blood pressure levels, from 127.2 mmHg in 1985 to 124.6 mmHg in 2008, a relative decrease of 2%. After accounting for the 3185 fewer deaths attributable to primary prevention with hypertension treatments, we estimated that approximately 10,365 CHD deaths were prevented or postponed due to reductions in mean blood pressure from life-style and dietary changes from 1995 to 2008, representing some 29% of the overall reduction in CHD mortality (Figure 1, Table 2).

Reductions in smoking prevalence in men (-46%) and in women (-27%), also explained approximately 9725 fewer CHD deaths overall.

Population mean cholesterol did not change significantly in Turkey between 1995 and 2008, a 0.02% increase in the population mean cholesterol levels resulted in some 355 deaths. Statin treatments for primary prevention in people with high cholesterol prevented or postponed approximately 725 deaths.

The effects of a decrease in physical inactivity prevalence and increase in fruit and vegetable consumption prevented or postponed approximately 1920 and 2375 deaths representing some 5% and 7% of the total fall in deaths respectively (Table 2).

However, there were adverse trends in diabetes prevalence and BMI levels between 1995 and 2008. Diabetes prevalence increased from 14.5 to 17.5% in men and 16.4% to 19.9% in women (20% and 17% relative increases) from 1995 to 2008, which resulted in approximately 1865 deaths in men and 3220 additional deaths in women representing some 14% of the total deaths. Increases in mean BMI (7.0% in men and 5.0% in women) generated approximately 3790 additional deaths, which effectively represents an additional 10% *(minimum 6.0% and maximum 16.2%)* to total CHD deaths *(Table *2*).*

### Sensitivity analysis and proportional contributions to the decrease in deaths

Sensitivity analyses suggested some uncertainty surrounding the risk factor best estimate contribution of 42%, with a range from 33% to 54% (Tables 1 and 2). There was a larger uncertainty in our estimates of the treatment contribution (best estimate 47% - minimum15%, maximum 99%). However, the proportional contributions of specific treatments and risk factor changes to the overall decrease of CHD mortality in Turkey between 1995 and 2008 remained relatively consistent, irrespective of whether best, minimum or maximum estimates were used (Tables 1 and 2).

## Discussion

In Turkey, CHD mortality rates fell by 31% between 1995 and 2008 which is similar to the falls reported in affluent Western countries, including England and Wales [7], Ireland [30], Sweden [50] and Italy [51], since the 1980s but occurring later in Turkey.

Approximately 47% of this mortality fall was attributable to the combined effects of modern cardiological treatments and almost 42% was attributable to reduction in major risk factors, particularly smoking and blood pressure. In the previous modelling studies more than half of the fall was explained by risk factor reductions in England and Wales [7], Ireland [30], in Sweden [50], Italy [51]. Modern cardiological treatments together prevented or postponed approximately 16,700 deaths in 2008 in Turkey. Irrespective of whether best, minimum or maximum estimates were used, the most substantial contributions came from secondary prevention and angina treatments.

Revascularisation from CABG surgery and angioplasty together accounted for barely 4% of the total mortality fall, much as in the USA [24] and other countries [7]. This represents a quite small contribution, particularly when considering the large financial and political resources being consumed [52].

Thrombolysis, likewise, only accounted for approximately one fifth of the deaths prevented in the initial treatments for acute myocardial infarction. Aspirin and cardiopulmonary resuscitation contributed far more, similar to other studies [7].

Furthermore, treating angina patients with aspirin in the community prevented almost four times as many deaths as treating unstable angina patients in hospitals, mainly reflecting the far greater numbers of patients eligible for this treatment (Table 1).

Treatment uptake levels were often poor, especially for beta blockers and ACE inhibitors for secondary prevention (Table 1). Earlier work in the UK suggested that if 80% of eligible patients had received appropriate therapy, approximately 30,000 additional deaths might have been prevented or postponed each year in the UK [53]. The same powerful principle would clearly apply in Turkey.

Reductions in the major risk factors between 1995 and 2008 accounted for approximately 15100 fewer deaths in Turkey in 2008. The biggest single contribution, 38%, reflected a 2.74 mmHg absolute decline in systolic blood pressure. The improvements in blood pressure are consistent with improvements in diet rather than treatment. Turkish diet is traditionally rich in fresh fruit and vegetables, legumes and unsaturated fat. Since the socioeconomic improvements in the 1990s, more people are now able to afford fresh fruit and vegetables rather than consuming preserved food that contain substantial salt. However, a recent national survey suggests that daily salt consumptions still very high, approximately 18 gr per day [54]. Traditionally, salt is widely used in pickles, olives, dairy products and bread which together constitute a major part of the Turkish diet. Future population based salt reduction strategies may achieve substantial further blood pressure reductions.

Almost 27% of the mortality fall came from a 41% relative reduction (from 26.5% to 16%) in male smoking. However, smoking prevalence did not change significantly in women. The National Tobacco Control Programme which began in 2008, may further accelerate this decreasing trend in Turkey [55]. The programme aims to increase awareness of the damaging health effects of smoking, together with the government anti-smoking measures including taxation, banning smoking in the public places, intensified anti-smoking campaigns, and the banning of advertising [55].

The adverse trends in obesity and diabetes together contributed over 9000 additional deaths in 2008. This therefore cancelled out much of the benefits attained by the decreases in blood pressure and smoking prevalence during the same period. Obesity and diabetes trends are alarming both in men and women in Turkey [12,13] with major concerns about future continuing deteriorations [56]. New diabetes and obesity control programs were prepared for the period of 2010–2014 [57,58] but these will need to be implemented aggressively to have any benefit.

Population mean cholesterol did not change significantly in Turkey between 1995 and 2008. Stable trends or only small decreases in cholesterol levels were also observed in several other Eastern European countries including Tunisia and Iran [19].

### Modelling strengths and limitations

Models are potentially useful tools for policy development. They integrate and simultaneously consider huge amounts of data from many different sources. On the other hand, they are very dependent on the quality and extent of data available on CHD risk factor trends and treatment uptakes [59,60].

The data used in the Turkish IMPACT model was generally of good quality. Mortality data was obtained from the Statistical Institute of Turkey (TURKSTAT) which has a long experience of death registry since 1930 [1]. Although the cause of death statistics were based on only urban area and the rates were estimated assuming similar death patterns exist in rural population, our estimates for year 2000 were similar with National Burden of Disease Study. The declining CHD mortality trend starting from the mid 1990s is thought to be real since there were no operational changes in the death reporting system over that time period. Data quality indicators such as the proportion of ill defined codes (symptoms/ senility or other heart disease) have remained consistent, which also supports this finding. During the entire period (1995to 2008) the proportion of senility, symptoms and other heart disease codes were relatively stable in men and women (ranging from 43-53% and 52-63%, respectively) (Data are available upon request from the authors). The demographic information was obtained from the census data that covered whole country, the risk factor trends were obtained from national epidemiological studies. Treatment uptake data was obtained from multicentre national studies (EUROASPIRE III [26], PREMISE [27]) and from a hospital based surveys conducted in recent years [43,61,62]. Certain assumptions were therefore taken to fill in the gaps for missing information including specific patient groups’ data. These assumptions are systematically detailed in the Additional file 1, and were supported by local expert opinions and literature from the region and included in the sensitivity analysis. In the Turkish IMPACT model, we assessed the potential maximum and minimum plausible effects of these factors using rigorous sensitivity analyses which systematically examined the influence of these uncertainties and the assumptions used in the studies [49].

Our study has several potential limitations. The model included only those adults aged 35 to 84 years, because of very limited data in older age groups. The efficacy data were derived from clinical trials and may have overestimated effectiveness in usual clinical practice. We considered only deaths from CHD, and ignored “competing causes” such as cancer. However, the reductions in smoking would actually have decreased deaths from lung cancer and some other cancers [63]. Finally, the lag times were not explicitly considered in this model assuming that substantial mortality reduction occurs within 1 to 4 years of quitting smoking or reducing cholesterol [64-66].

This is the first comprehensive modelling study that examines the impact of changes in population risk factors and effective treatments on CHD mortality trends in Turkey. The Ministry of Health developed policy documents and action plans to tackle the growing NCD problem [55,57,58,67]. The Ministry of Health also coordinated the development of national guidelines for CHD, hypertension and diabetes control. This model can be a useful tool to explore potential benefits of implementing certain strategies to prevent the future CHD.

## Conclusions

Coronary heart disease mortality in Turkey fell by more than 30 percent between 1995 and 2008. Approximately half of this decline was explained by treatments. However, although changes in major risk factors, mainly smoking and blood pressure potentially explained over half of the decline, much of these benefits were then negated by worrying increases in obesity and diabetes. Our findings thus emphasize the value of primary prevention strategies targeting the whole population including healthy diet and tobacco control, plus evidence-based medical treatments for our patients in Turkey.

## Abbreviations

CHD: Coronary heart disease; NCDs: Non-communicable diseases; TURDEP: Turkish diabetes epidemiology study; PCI: Percutaneous coronary intervention; CABG: Coronary artery bypass surgery; ACE-inhibitors: Angiotensin converting enzyme inhibitors; ARBs: Angiotensin-receptor-blockers; ICD: International classification of diseases; MoH: Ministry of health; BMI: Body mass index; AMI: Acute myocardial infarction.

## Competing interests

The authors declare that they have no competing interests.

## Authors’ contributions

BU conceived the study question, interpreted the findings, and wrote the first dart of the article. KS, GG, HA, DA, HŞ, SD, YD, ÖA contributed to the data collection and critical appraisal of data sources, analysis and interpretation of findings, and drafting of the manuscript. KB,MOF,SC and JC contributed to drafting the manuscript, analysis, interpretation of findings, critical revision, and supervision of the study. All authors read and approved the final manuscript.

## Pre-publication history

The pre-publication history for this paper can be accessed here:

http://www.biomedcentral.com/1471-2458/13/1135/prepub

## Supplementary Material

Additional file 1 **The Turkish IMPACT Model. Table S1.** Main Data Sources Populating the Turkey IMPACT Model. **Table S2.** Treatment Uptake Data Sources. **Table S3.** Age-Specific Case Fatality Rates for Each Patient Group. **Table S4.** Clinical Efficacy of Interventions: Relative Risk Reductions Obtained From Meta-Analyses, and Randomized Controlled Trials. **Table S5.** Specific Beta Coefficients for Major Risk Factors. Click here for file

## References

[B1] Turkish Statistical Institute (Turkstat) website Statistics by theme/Population and demography/vital statistics/statistical tables and dynamic search/death statisticshttp://tuikapp.tuik.gov.tr/adnksdagitapp/adnks.zul

[B2] Koc I Eryurt MA Adalli T Seckiner P Türkiye’nin demografik dönüşümü Doğurganlık, Aile Planlaması, Anne-Çocuk Sağlığı ve Beş Yaş Altı Ölümlerdeki Değişimler: 1968–2008 2010 Ankara: Hacettepe Üniversitesi Nüfus Etütleri Enstitüsü http://www.hips.hacettepe.edu.tr/TurkiyeninDemografikDonusumu_220410.pdf

[B3] Ministry of Health National Burden of Disease and Cost Effectiveness Study Burden of Disease Final Report, 2004 (in Turkish) 2004 Ankara: Refik Saydam Hygiene Center Presidency http://ekutuphane.tusak.gov.tr/kitaplar/turkiye_hastalik_yuku_calismasi.pdf

[B4] British Heart Foundation Coronary Heart Disease Statistics 2010 2010 Oxford http://www.bhf.org.uk/publications/view-publication.aspx?ps=1001546

[B5] Dinc G Gerceklioglu G Sozmen K Arık H Unal B Decreasing trends in cardiovascular mortality in Turkey between 1995 and 2008 BMC Public Health 2013 13 896 10.1186/1471-2458-13-896 24079269 PMC3850640

[B6] Capewell S Ford ES Croft JB Critchley JA Greenlund KJ Labarthe DR Cardiovascular risk factor trends and potential for reducing coronary heart disease mortality in the United States of America Bull World Health Organ 2010 88 2 120 130 10.2471/BLT.08.057885 20428369 PMC2814476

[B7] Unal B Critchley J Capewell S Explaining the decline in coronary heart disease mortality in England and Wales, 1981–2000 Circulation 2004 109 1101 1107 10.1161/01.CIR.0000118498.35499.B2 14993137

[B8] Laatikainen T Critchley J Vartiainen E Salomaa V Ketonen M Capewell S Explaining the decline in coronary heart disease mortality in Finland between 1982 and 1997 Am J Epidemiol 2005 162 8 764 773 10.1093/aje/kwi274 16150890

[B9] Onat A Senocak MS Surdum-Avci G Ornek E Prevalence of coronary heart disease in Turkish adults IntJCardiol 1993 39 1 23 31 10.1016/0167-5273(93)90293-p 8407004

[B10] Altun B Arici M Nergizoglu G Derici U Karatan O Turgan C Sindel S Erbay B Hasanoglu E Caglar S Prevalence, awareness, treatment and control of hypertension in Turkey (the PatenT study) in 2003 J Hypertens 2005 23 10 1817 1823 10.1097/01.hjh.0000176789.89505.59 16148604

[B11] Arici M Turgan C Altun B Sindel S Erbay B Derici U Karatan O Erdem Y Hasanoglu E Caglar S Hypertension incidence in Turkey (HinT): a population-based study J Hypertens 2010 28 2 240 244 10.1097/HJH.0b013e328332c36b 19809361

[B12] Satman I Yilmaz T Sengul A Salman S Salman F Uygur S Bastar I Tutuncu Y Sargin M Dinccag N Population-based study of diabetes and risk characteristics in Turkey: results of the turkish diabetes epidemiology study (TURDEP) Diabetes Care 2002 25 9 1551 1556 10.2337/diacare.25.9.1551 12196426

[B13] Satman I Omer B Tutuncu Y Kalaca S Gedik S Dinccag N Karsidag K Genc S Telci A Canbaz B Turker F Yilmaz T Cakir B Tuomilehto J Twelve-year trends in the prevalence and risk factors of diabetes and prediabetes in Turkish adults Eur J Epidemiol 2013 doi:10.1007/s10654-013-9771-5 10.1007/s10654-013-9771-5 PMC3604592 23407904

[B14] Ergor G Soysal A Sozmen K Unal B Uçku R Kılıç B Balcova Heart Study- Rationale and Methodology of the Turkish Cohort Int J Public Health 2011 57 3 535 542 21987028 10.1007/s00038-011-0309-x

[B15] Erem C Hacihasanoglu A Deger O Kocak M Topbas M Prevalence of dyslipidemia and associated risk factors among Turkish adults: Trabzon lipid study Endocrine 2008 34 1–3 36 51 19003544 10.1007/s12020-008-9100-z

[B16] Kozan O Oguz A Abaci A Erol C Ongen Z Temizhan A Celik S Prevalence of the metabolic syndrome among Turkish adults Eur J Clin Nutr 2007 61 4 548 553 17119546 10.1038/sj.ejcn.1602554

[B17] Sanisoglu SY Oktenli C Hasimi A Yokusoglu M Ugurlu M Prevalence of metabolic syndrome-related disorders in a large adult population in Turkey BMC Public Health 2006 6 92 10.1186/1471-2458-6-92 16606462 PMC1458328

[B18] Danaei G Finucane MM Lin JK Singh GM Paciorek CJ Cowan MJ Farzadfar F Stevens GA Lim SS Riley LM National, regional, and global trends in systolic blood pressure since 1980: systematic analysis of health examination surveys and epidemiological studies with 786 country-years and 5.4 million participants Lancet 2011 377 9765 568 577 10.1016/S0140-6736(10)62036-3 21295844

[B19] Farzadfar F Finucane MM Danaei G Pelizzari PM Cowan MJ Paciorek CJ Singh GM Lin JK Stevens GA Riley LM National, regional, and global trends in serum total cholesterol since 1980: systematic analysis of health examination surveys and epidemiological studies with 321 country-years and 3.0 million participants Lancet 2011 377 9765 578 586 10.1016/S0140-6736(10)62038-7 21295847

[B20] The Ministry of Health of Turkey Global Adult Tobacco Survey Turkey Report 2010 Ankara http://www.havanikoru.org.tr/dosya/Docs_Tutun_Dumaninin_Zararlari/GATS.pdf

[B21] Danaei G Finucane MM Lu Y Singh GM Cowan MJ Paciorek CJ Lin JK Farzadfar F Khang YH Stevens GA National, regional, and global trends in fasting plasma glucose and diabetes prevalence since 1980: systematic analysis of health examination surveys and epidemiological studies with 370 country-years and 2.7 million participants Lancet 2011 378 9785 31 40 10.1016/S0140-6736(11)60679-X 21705069

[B22] Finucane MM Stevens GA Cowan MJ Danaei G Lin JK Paciorek CJ Singh GM Gutierrez HR Lu Y Bahalim AN National, regional, and global trends in body-mass index since 1980: systematic analysis of health examination surveys and epidemiological studies with 960 country-years and 9.1 million participants Lancet 2011 377 9765 557 567 10.1016/S0140-6736(10)62037-5 21295846 PMC4472365

[B23] Capewell S Beaglehole R Seddon M McMurray J Explaining the decline in Coronary Heart Disease Mortality in Auckland, New Zealand between 1982 and 1993 Circulation 2000 102 1511 1516 10.1161/01.CIR.102.13.1511 11004141

[B24] Ford ES Ajani UA Croft JB Critchley JA Labarthe DR Kottke TE Giles WH Capewell S Explaining the decrease in U.S. deaths from coronary disease, 1980–2000 N Engl J Med 2007 356 23 2388 2398 10.1056/NEJMsa053935 17554120

[B25] Capewell S O'Flaherty M What explains declining coronary mortality? Lessons and warnings Heart 2008 94 9 1105 1108 10.1136/hrt.2008.149930 18703684

[B26] Tokgözoğlu LKE Erol C Ergene O EUROASPIRE III Turkey Study Group: [EUROASPIRE III: a comparison between Turkey and Europe] TurkKardiyolDernArs 2010 38 3 164 172 20675993

[B27] Mendis S Abegunde D Yusuf S Ebrahim S Shaper G Ghannem H Shengelia B WHO study on Prevention of REcurrences of Myocardial Infarction and StrokE (WHO-PREMISE) Bull World Health Organ 2005 83 11 820 829 16302038 PMC2626468

[B28] Capewell S Morrison CE McMurray JJ Contribution of modern cardiovascular treatment and risk factor changes to the decline in coronary heart disease mortality in Scotland between 1975 and 1994 Heart 1999 81 4 380 386 10092564 10.1136/hrt.81.4.380 PMC1729021

[B29] Critchley J Liu J Zhao D Wei W Capewell S Explaining the increase in coronary heart disease mortality in Beijing between 1984 and 1999 Circulation 2004 110 1236 1244 10.1161/01.CIR.0000140668.91896.AE 15337690

[B30] Bennett K Kabir Z Unal B Shelley E Critchley J Perry I Feely J Capewell S Explaining the recent decrease in coronary heart disease mortality rates in Ireland, 1985–2000 J Epidemiol Community Health 2006 60 4 322 327 10.1136/jech.2005.038638 16537349 PMC2566167

[B31] Note by Turkish Statistical Institute New method for 2010 population and housing census of Turkey considerations about data quality and coverage http://www.unece.org/fileadmin/DAM/stats/documents/ece/ces/ge.41/2008/sp.3.e.pdf

[B32] Razum O Akgun S Tezcan S Cardiovascular mortality patterns in Turkey: what is the evidence? Soz Praventivmed 2000 45 1 46 51 10.1007/BF01358998 10743029

[B33] Appendices for IMPACT CHD Mortality Model http://www.liv.ac.uk/PublicHealth/sc/bua/impact.html

[B34] Erem C Yildiz R Kavgaci H Karahan C Deger O Can G Telatar M Prevalence of diabetes, obesity and hypertension in a Turkish population (Trabzon city) Diabetes Res Clin Pract 2001 54 3 203 208 10.1016/S0168-8227(01)00320-5 11689275

[B35] Tezcan S Altintas H Sonmez R Akinci A Dogan B Cakir B Bilgin Y Klor HU Razum O Cardiovascular risk factor levels in a lower middle-class community in Ankara, Turkey Trop Med Int Health 2003 8 7 660 667 10.1046/j.1365-3156.2003.01057.x 12828550

[B36] Republic of Turkey Distribution of Inpatiens by 150 Selected Diseases in Turkey. Health Statistics Year Book. Kalkan Matbaacılık 2011 Ankara http://www.saglik.gov.tr/TR/dosya/1-72577/h/saglikistatistikleriyilligi2010.pdf

[B37] Aslan BU Karcioglu O Aslan O Ayrik C Kulac E Guneri S [Does the short-term mortality differ between men and women with first acute myocardial infarction?] AnadoluKardiyolDerg 2002 2 4 284 290 12460822

[B38] Badillioglu O Unal B Ucku R Five-year incidence of coronary heart disease and risk factors in Güzelbahçe, İzmir Turk J Public Health 2011 9 3 129 132

[B39] Aslan BU Karcioglu O Aslan O Ayrik C Kulac E Guneri S Does the short-term mortality differ between men and women with first acute myocardial infarction? Anadolu Kardiyol Derg 2002 2 4 284 290 12460822

[B40] Turkish Statistical Institute Health Survey 2008 Report 2010 Ankara: Turkish Statistical Institute Printing Division

[B41] Turkish Statistical Institute Health Survey 2010 Report 2012 Ankara: Turkish Statistical Institute Printing Division

[B42] Unal B Sozmen K Ucku R Ergor G Soysal A Baydur H Meseri R Simsek H Gerceklioglu G Doganay S High prevalence of cardiovascular risk factors in a Western urban Turkish population: a community-based study Anadolu Kardiyol Derg 2013 13 9 17 23070631 10.5152/akd.2013.002

[B43] Ildızlı MKM Yavuzgil O Hasdemir C Gürgün C Kültürsay H To what extent are we applying current medical treatment approaches in coronary artery disease? Arch Turk Soc Cardiol 2004 32 542 549

[B44] Mant J Hicks N Detecting differences in quality of care: the sensitivity of measures of process and outcome in treating acute myocardial infarction BMJ 1995 311 7008 793 796 10.1136/bmj.311.7008.793 7580444 PMC2550793

[B45] Capewell S Chalmers J MacIntyre K Social gradients in AMI mortality rates rates (per 100,000) in the Scottish population 1986–1995 (quintiles of deprivation in men) 2000 Personal communication

[B46] Capewell S MacIntyre K Stewart S Chalmers JW Boyd J Finlayson A Redpath A Pell JP McMurray JJ Age, sex, and social trends in out-of-hospital cardiac deaths in Scotland 1986–95: a retrospective cohort study Lancet 2001 358 9289 1213 1217 10.1016/S0140-6736(01)06343-7 11675057

[B47] Capewell S Livingston BM MacIntyre K Trends in case-fatality in 117 718 patients admitted with acute myocardial infarction in Scotland Eur Heart J 2000 21 22 1833 1840 10.1053/euhj.2000.2318 11052855

[B48] Nichol MB Venturini F Sung JC A critical evaluation of the methodology of the literature on medication compliance Ann Pharmacother 1999 33 5 531 540 10.1345/aph.18233 10369613

[B49] Briggs A Economics notes: handling uncertainty in economic evaluation BMJ 1999 319 7202 120 10.1136/bmj.319.7202.120 10398643 PMC1116199

[B50] Bjorck L Rosengren A Bennett K Lappas G Capewell S Modelling the decreasing coronary heart disease mortality in Sweden between 1986 and 2002 Eur Heart J 2009 30 9 1046 1056 10.1093/eurheartj/ehn554 19141562

[B51] Palmieri L Bennett K Giampaoli S Capewell S Explaining the decrease in coronary heart disease mortality in Italy between 1980 and 2000 Am J Public Health 2010 100 4 684 692 10.2105/AJPH.2008.147173 19608958 PMC2836342

[B52] Liu JL Maniadakis N Gray A Rayner M The economic burden of coronary heart disease in the UK Heart 2002 88 6 597 603 10.1136/heart.88.6.597 12433888 PMC1767465

[B53] Capewell S Unal B Critchley JA McMurray JJ Over 20,000 avoidable coronary deaths in England and Wales in 2000: the failure to give effective treatments to many eligible patients Heart 2006 92 4 521 523 16537767 10.1136/hrt.2004.053645 PMC1860878

[B54] Erdem Y Arici M Altun B Turgan C Sindel S Erbay B Derici U Karatan O Hasanoglu E Caglar S The relationship between hypertension and salt intake in Turkish population: SALTURK study Blood Press 2010 19 5 313 318 10.3109/08037051003802541 20698734

[B55] Ministry of Health of Turkey National Tobacco Control Programme and Action Plan of Turkey 2008–2012 2008 Ankara http://www.tkd-online.org/PDFs/tobacco_plan_en.pdf

[B56] Altun D Sozmen K Kalaca S Unal B How does obesity prevalence change in Turkey? -Oral presentation14th National Public Health 2011 Trabzon, Turkey: Congress Book S046

[B57] Ministry of Health of Turkey Obesity Prevention and Control Program of Turkey (2010–2014) 2010 Ankara: Kuban Matbaacilik Yayincilik http://www.beslenme.gov.tr/content/files/home/obesity_prevention_and_control_program_of_turkey_2010_2014.pdf

[B58] Ministry of Health of Turkey Turkey diabetes prevention and control program 2011–2014 2011 Ankara: Anıl Matbaası http://www.saglik.gov.tr/HM/dosya/1-71375/h/turkiye-diyabet-onleme-ve-kontrol-programi.pdf

[B59] Critchley JA Capewell S Why model coronary heart disease? Eur Heart J 2002 23 2 110 116 10.1053/euhj.2001.2681 11785992

[B60] Unal B Capewell S Critchley JA Coronary heart disease policy models: a systematic review BMC Public Health 2006 6 213 10.1186/1471-2458-6-213 16919155 PMC1560128

[B61] Simsek H Demiral Y Aslan O Unal B Treatment uptake levels in the coronary heart disease patients at hospital discharge Int J Cardiol 2011 147 S156 S156

[B62] Sonmez K Akcay A Akcakoyun M Demir D Elonu OH Onat O Duran NE Gencbay M Degertekin M Turan F Distribution of therapeutic procedures and choice of drug therapies in patients with angiographically confirmed coronary artery disease AnadoluKardiyolDerg 2002 2 1 18 23 AXVI 12101790

[B63] Fichtenberg CM Glantz SA Association of the California Tobacco Control Program with Declines in Cigarette Consumption and Mortality from Heart Disease N Engl J Med 2001 343 24 1772 1777 11114317 10.1056/NEJM200012143432406

[B64] Critchley J Capewell S Smoking cessation for the secondary prevention of coronary heart disease Cochrane Database Syst Rev 2004 1 CD003041 10.1002/14651858.CD003041.pub2 14974003

[B65] Law MR Wald NJ Thompson SG By how much and how quickly does reduction in serum cholesterol concentration lower risk of ischaemic heart disease? BMJ 1994 308 6925 367 372 10.1136/bmj.308.6925.367 8043072 PMC2539460

[B66] Capewell S O'Flaherty M Mortality falls can rapidly follow population-wide risk factor changes Lancet 2011 378 9793 752 753 10.1016/S0140-6736(10)62302-1 21414659

[B67] The Ministry of Health of Turkey Prevention and Control Program for Cardiovascular Diseases: Strategic Plan and Action Plan for the Risk Factors 2009 Ankara http://sbu.saglik.gov.tr/Ekutuphane/kitaplar/t4.pdf

