# Supplementary material for: Explaining the decline in coronary heart disease mortality in Turkey between 1995 and 2008
Source: BMC Public Health. 2013 Dec 5;13:1135. doi: 10.1186/1471-2458-13-1135 (PMC4234124; doi:10.1186/1471-2458-13-1135)
Supplement: Additional file 1 — The Turkish IMPACT Model. Table S1. Main Data Sources Populating the Turkey IMPACT Model. Table S2. Treatment Uptake Data Sources. Table S3. Age-Specific Case Fatality Rates for Each Patient Group. Table S4. Clinical Efficacy of Interventions: Relative Risk Reductions Obtained From Meta-Analyses, and Randomized Controlled Trials. Table S5. Specific Beta Coefficients for Major Risk Factors. [file 1471-2458-13-1135-S1.docx]

**Turkish IMPACT Model Technical Appendix**

**Supplementary Online Content**

**Appendix. The Turkish IMPACT Model**

**Table 1.** Main Data Sources Populating the Turkey IMPACT Model

**Table 2.** Treatment Uptake Data Sources

**Table 3.** Age-Specific Case Fatality Rates for Each Patient Group

**Table 4.** Clinical Efficacy of Interventions: Relative Risk Reductions Obtained From Meta-Analyses, and Randomized Controlled Trials

**Table5.** Specific Beta Coefficients for Major Risk Factors

**Appendix. The Turkish IMPACT Model**

We evaluated the over 35 years of age population of Turkey using an updated version of the IMPACT model. This is a cell-based model, constructed using Microsoft Excel, which integrates available country-specific epidemiological data to explain an observed decrease in CHD mortality over a period of time. The tables included in this supplementary appendix document provide details about these methods. This model has been validated in Europe, New Zealand, China and the United States[[1-5](#_ENREF_1)].

**Population data**

The population data were obtained from population census for 1995 and address based population registry system for year 2008. Census was conducted by the Turkish Statistical Institute (TurkStat) using standardised methodology in every 5 years between 1935 and 2000. TurkStat is also responsible for address based population registry which is updated continuously[[6](#_ENREF_6)].

**Changes in mortality rates from CHD in Turkey from 1995 to 2008**

Mortality data was obtained from Turkish Statistical Institute (TurkStat) for the years 1995 up to 2008[[7](#_ENREF_7)]. Total number of deaths was inflated by 15% to account low coverage. Number of CHD deaths was estimated by allocating %50 of “other heart disease” to CHD deaths based on expert opinion from National Burden of Disease Project[[8](#_ENREF_8)]. Mortality rates from CHD were calculated using the underlying cause of death; International Classification of Diseases (ICD)-9 codes 410-414, 428, 429.2 and ICD-10 codes I20-I25, I50. As we were only interested in deaths from coronary artery disease we only included heart failure deaths with the code of I50 which was a result of ischaemic cardiomyopathy (See eTable 1 for details).

**Expected and observed number of deaths from CHD**

The primary output of the IMPACT model was the number of deaths prevented or postponed in 2008 due to the reduction in CHD mortality rates. This was calculated as the difference between the observed 2008 CHD deaths and the expected CHD deaths in 2008 if 1995 mortality rates remained constant over the time. The expected number of CHD deaths was calculated by multiplying age and gender specific mortality rates in 1995 by the population size for each 10-year age-gender stratum in 2008.

**Patient Groups**

The treatment arm of the Model includes the following groups:

• Hospitalized patients with an acute myocardial infarction (AMI) in the last year (2008)

• Hospitalized patients with Unstable Angina Pectoris (AP) in the last year (2008)

• Community-dwelling patients who have survived an AMI in the past 5 years (2004-2008)

• Community-dwelling patients with chronic Angina who have undergone revascularisation procedure Coronary Artery Bypass Grafting (CABG), or a Percutaneous Coronary Intervention (PCI), within the last year for chronic Angina.

•Community-dwelling patients with chronic stable coronary artery disease (no revascularisation and/or previous MI)

• Hospitalized patients with heart failure within the last year,

• Community-dwelling patients with heart failure,

• Hypertensive patients eligible for pharmacological therapy and have not suffered any of the above events.

• Hypercholesterolemic patients eligible for cholesterol lowering therapy (Statin) and have not suffered any of the above events[[9](#_ENREF_9)].

**Data sources for Patient Groups**

**Hospital patient groups** were calculated based on the number of admissions to hospitals obtained from Ministry of Health separately for AMI, AP and CHF.

**Number of patients with chronic angina** was calculated based on National Burden Of Disease and Cost Effectiveness Project (NBD-CEP) findings[[8](#_ENREF_8)] and Health Survey 2008[[10](#_ENREF_10)]. Both studies provided prevalence of chronic angina in the community.

**Number of patients with heart failure living in the community**: was calculated based on results of a national heart failure survey titled HAPPY Study[[11](#_ENREF_11)].

**Statins for primary prevention** was calculated based on Balçova Heart Study[[12](#_ENREF_12), [13](#_ENREF_13)] which provides estimates of hypercholesterolemia patients receiving medications.

**Antihypertensive medication** was estimated based on Balçova Heart Study[[12](#_ENREF_12), [13](#_ENREF_13)] which provides estimates of patients with Hypertension receiving medications.

**Secondary prevention post MI**  was calculated based on the estimated number of AMI patients in 2008 using incidence estimates from cohort studies[[14](#_ENREF_14), [15](#_ENREF_15)]. We assumed 10% fewer in each preceding year and 10% case fatality rate every year. These assumptions were supported by local expert opinions. The estimated number was 433755.

**Secondary prevention following CABG/PTCA:** was calculated based on the estimated number of AMI patients in 2008 using incidence estimates from cohort studies[[14](#_ENREF_14), [15](#_ENREF_15)]. We assumed 20% of AMIs get CABG based on a follow-up study[[16](#_ENREF_16)] and expert opinion. Half the estimated number was assumed as MI survivals and the final number included in the model was 103295. Similar calculation was done for PTCA.

**Potential overlaps between patient groups**

There are potential overlaps between patient groups (meaning that one person may belong to more than one patient group at the same time). Hospital patient groups were selected based on one-year case fatality and overlapping between groups was avoided. Community patients groups were calculated based on the numbers of hospitalized patients groups with assumptions based on the literature.

**Treatments**

Data on treatment levels for angina pectoris, AMI, PCI, CABG and heart failure was collected through patient records in Dokuz Eylul University Hospital. Patients’ records with ICD 10 codes of I20-25, I50, I60, I61, I63, I64, and I70-I78 in 2008 were provided by the Hospital Data Management Unit. The medical history of each patient was screened from the hospital discharge reports. Double records were checked and if there is more than one record for a person only the first admission was recruited in the study. Patients were excluded if their ICD code was not supported by the medical history in their records. Emergency records were also excluded since they did not provide standard discharge reports. After double entries were removed from the list, there were 1355 inpatient records. Findings were compared with the literature[[17](#_ENREF_17)] and made adjustment if necessary with an expert from Cardiology Department(Prof.Dr.Özgür Aslan)

Balcova Heart Study baseline survey provided treatment uptake levels for heart diseases. All the treatments were coded based on ATC system[[12](#_ENREF_12)]. Database is available to the authors and the treatments were regrouped based on the needs of the model.

Results of previous studies focused on secondary prevention treatments for CHD such as PREMISE and EUROASPIRE-III surveys included data from Turkey. PREMISE Study was done in 2002, included 1034 CHD and SVD patients from Eskişehir[[18](#_ENREF_18)]. EUROASPIRE III collected data on secondary prevention treatments from 669 patients from 17 centers[[17](#_ENREF_17)].

Treatment uptake for patients groups in the community was collected from the community based Balcova Heart Study [[12](#_ENREF_12)] .

For each of the groups, we estimated the number of DPPs that were attributable to various treatments. All treatments of interest are listed in Table 2.

The deaths prevented or postponed associated with a specific CHD treatment within a disease subgroup was estimated by taking the product of the number of people in the subgroup (eTable 1), the proportion of those patients who received a particular treatment (eTable 2), the 1 year mortality rate (eTable 3), Supplementary Appendix), and the relative risk reduction attributed to that specific treatment based on the published literature (eTable 4). We assumed that compliance defined as the proportion of patients prescribed medications on therapeutic doses of medication, was 100% among hospital patients, 70% among symptomatic community patients and 50% in asymptomatic individuals taking statins or anti-hypertensives for primary prevention.

All these assumptions were tested in subsequent sensitivity analyses.

**EXAMPLE 1: estimation of DPPs from a specific treatment**

In Turkey in 2008, 13086 men aged 55-64 were hospitalized with AMI. Utilization of aspirin was 98%[[19](#_ENREF_19)], Efficacy of aspirin[[20](#_ENREF_20)] is 15%, 1-year case-fatality rate was 5.4[[21](#_ENREF_21)].

The deaths prevented or postponed (DPPs) was calculated as:

***Patient numbers x treatment uptake x relative mortality reduction x one-year case fatality***

***=*** 13086 x 98% x 15% X 5.4% = 104 deaths prevented or postponed.

**Risk factors**

The IMPACT model calculates the DPPs associated with changes in CHD risk factors, including smoking, total cholesterol, systolic blood pressure, body mass index, diabetes mellitus, and physical inactivity. Data sources are shown in eTable 1.

In the model we used findings from five nationwide and one local study that enabled us to evaluate the trends between 1995 and 2008. Linear interpolation was used when data were not available for a given year.

Smoking and physical inactivity were self reported in data sources. Trends in mean blood pressure values, mean total cholesterol values, and prevalence of diabetes, smoking, physical inactivity and fruit vegetable consumption were separately checked with the other local and nationwide study findings.

To assess the validity of these assumptions, we compared the reductions in systolic blood pressure and total cholesterol over the time horizon of the Turkey IMPACT model to those observed in previous IMPACT models. Further, trends and age gradients were compared with neighbouring countries.

Two approaches were used to calculate DPPs, the **regression approach** and the **population-attributable risk factor (PARF) approach.** The regression approach was used for continuous variables (systolic blood pressure, total cholesterol, and body mass index). The number of expected deaths from CHD occurring in 2008 (the end year) was multiplied by the absolute change in risk factor prevalence, and by a regression coefficient quantifying the change in CHD mortality that would result from the change in risk factor level. Natural logarithms were used, assuming a log-linear relationship between changes in risk factor levels and mortality.

**EXAMPLE 2: estimation of DPPs from risk factor change using regression method:**

***Mortality fall due to reduction in systolic blood pressure in women aged 55-64***

In 2008, there were 7896 CHD expected deaths (had 1995 mortality rates remained constant) among 2,607,696 women aged 55-64 years. Mean systolic blood pressure decreased by 5.6 mmHg (from 138.3 in 1998 to 132.7 mmHg in 2008). For every 20 mmHg reduction in systolic blood pressure, we estimated an age- and sex specific reduction in mortality of 50 percent. This generates a logarithmic coefficient of –0.035[[22](#_ENREF_22)].

The number of deaths prevented or postponed:

= (1-(EXP(coefficient*change))*expected deaths in 2008)

= (1-(EXP(-0.035*5.6))* 7896)

= 1420 DPPs

Data sources for the number of CHD deaths and risk factors are shown in eTable 1, and sources for the coefficients in eTable 5.

The PARF approach was used for categorical variables (smoking, diabetes, and physical inactivity). PARF was calculated as:

**(P x (RR-1)) / (P x (RR-1)) +1**

where P is the prevalence of the risk factor and RR is the relative risk for CHD mortality associated with that risk factor. DPPs were then estimated as the expected CHD deaths in 2008 multiplied by the difference in the PARF for 1995 and 2008.

**EXAMPLE 3: estimation of DPPs from risk factor change using PARF method**

The prevalence of diabetes among men aged 65-74 years was 25% in 1995 and 28% in 2008. Assuming a Relative Risk of 1.93[[23](#_ENREF_23)], the PARF was 0.17 in 1995 and 0.21 in 2008. The number of deaths attributable to the increase in diabetes prevalence from 1995 to 2008 was therefore:

(16261) * ( 0.28-0.25 ) = 299 DPPs

Data sources for the prevalence of risk factors and for the number of CHD deaths are shown in Table e1. The relative risks used in these PARF analyses were obtained from the INTERHEART study[[23](#_ENREF_23)], which provides independent RR values, adjusted for other major risk factors.

**Other Methodological Considerations**

**a. Systolic BP and Hyperlipidemia**

In order to separate the DPPs from pharmacological versus non-pharmacological primary prevention of hypertension and hyperlipidemia, we subtracted the age-gender specific DPP’s calculated in the treatment section (i.e. for primary hyperlipidemia and hypertension patient groups), from the DPP’s calculated in the risk factor section.

**b. Polypharmacy Issues**

There is a paucity of data on the efficacy of treatment combinations. Simply assuming that the efficacy of multiple treatments was additive would over-estimate the treatment effect; we therefore we used the Mant and Hicks method to estimate case-fatality reduction by polypharmacy for all treatments evaluated[[24](#_ENREF_24)]. This approach was subsequently endorsed by Yusuf[[25](#_ENREF_25)] and Law and Wald[[26](#_ENREF_26)]. This approach estimates a cumulative relative benefit as follows:

Relative Benefit = 1 - ((1-relative reduction in case-fatality rate for treatment A) X (1- relative reduction in case-fatality rate for treatment B) X ...X (1- relative reduction in case-fatality rate for treatment N).

**EXAMPLE 4: estimation of reduced benefit if patient taking multiple medications (Mant and Hicks approach)**

For AMI survivors, applying relative risk reductions (RRR) for aspirin, beta-blockers ACE inhibitors statins and rehabilitation then gives:

***Relative Benefit = 1 - [(1 –aspirin RRR) X (1 - beta-blockers RRR) X (1 - ACE inhibitors RRR) X (1- statins RRR) X (1- rehabilitation RRR)]***

= 1 - [(1- 0.15) X (1-0.23) X (1-0.20) X (1- 0.22) X (1- 0.26)]

= 1 - [(0.85) X (0.77) X (0.80) X (0.78) X (0.74)]

= 0.70 i.e. a 70% lower case fatality

**c. Sensitivity Analyses**

Because of the uncertainty surrounding many of the values, multi-way sensitivity analyses were performed[[27](#_ENREF_27)]. For each model parameter, a maximum and minimum plausible value was assigned using the 95% confidence intervals from the source documentation; if this was unavailable, we defined these limits as 20% above and below the best estimate. The maximum and minimum plausible values were fed in to the model generating maximum and minimum estimates for deaths prevented or postponed.

**Table 1. Main Data Sources for Populating the Turkish IMPACT Model**

| **Information** | **1995** | **2008** | **Comments** |
| --- | --- | --- | --- |
| Population Statistics | Turkish Statistical Institute[[6](#_ENREF_6)] | Turkish Statistical Institute[[6](#_ENREF_6)] | Age-gender specific population data were based on census for 1995 and Address Based Population Registration System for 2008. |
| Number of deaths by age and sex | Turkish Statistical Institute[[7](#_ENREF_7)] | Turkish Statistical Institute[[7](#_ENREF_7)] | Mortality data was available from the TURKSTAT (Statistical Institute of Turkey). Cause specific mortality data was available for urban settings which comprise approximately 60% of the total population. The other major limitation of the mortality data was coding inaccuracy. Ill defined codes were redistributed (ie 50% of “other heart disease” was distributed to ischaemic heart disease) |
| **Number of patients admitted yearly** |  |  |  |
| AMI |  | Hospital admission statistics. Turkey Statistical Institute[[28](#_ENREF_28)]. | Numbers were adjusted based on the incidence estimated using Framingham risk score[[29](#_ENREF_29)] and DISMOD calculations. |
| Acute Angina |  | Hospital admission statistics. Turkey Statistical Institute[[28](#_ENREF_28)]. |  |
| Heart failure |  | Hospital admission statistics. Turkey Statistical Institute[[28](#_ENREF_28)]. |  |
| **Number of patients treated yearly with** |  |  |  |
| CABG | Estimated based on survey results and expert opinion | |  |
| PCI | Estimated based on survey results and expert opinion | |  |
| Post-MI | Estimated based on survey results and expert opinion | |  |
| Community chronic angina |  | Health Survey 2010[[30](#_ENREF_30)] | Self reported rates checked with a cardiologist and compared with the literature |
| Community Heart Failure |  | HAPPY study[[11](#_ENREF_11)] | Based on a national sample of 4650 people |
| Hypertension (primary prevention) |  | National and regional surveys on hypertension[[12](#_ENREF_12), [31](#_ENREF_31)] | Balçova Heart Study dataset is available to the authors where the medications were coded based on ATC |
| Hyperlipidemia (primary prevention) |  | National and regional surveys[[32-34](#_ENREF_32)] |  |
| **Population Risk Factor Prevalence** |  |  |  |
| Current cigarette smoking | TURDEP-1[[35](#_ENREF_35)],  National Burden of Disease and Cost effectiveness Project[[8](#_ENREF_8)] | Adult Tobacco Survey[[36](#_ENREF_36)], Balçova Heart Study[[13](#_ENREF_13)], TURDEP-2[[33](#_ENREF_33)] | Linear interpolation was used for data points for each age band. TURDEP-2 findings were obtained by personal communication. |
| Systolic Blood Pressure | TURDEP-1[[35](#_ENREF_35)] and regional surveys[[37](#_ENREF_37)] | TURDEP-2[[33](#_ENREF_33)] |  |
| Total cholesterol | TEKHARF 1990[[38](#_ENREF_38), [39](#_ENREF_39)] | Balçova Heart Study[[13](#_ENREF_13)] and TURDEP-2[[33](#_ENREF_33)] |  |
| Physical inactivity | National Burden of Disease and Cost effectiveness Project[[8](#_ENREF_8)] | Balcova Heart Study 2010[[12](#_ENREF_12), [13](#_ENREF_13)] |  |
| BMI | TURDEP-1[[35](#_ENREF_35)] | Sanisoglu[[34](#_ENREF_34)] and TURDEP II Study[[33](#_ENREF_33)] |  |
| Diabetes | Surveys on diabetes epidemiology TURDEP-1[[35](#_ENREF_35)],  and Sanisoglu[[34](#_ENREF_34)] | TURDEP II Study[[33](#_ENREF_33)] |  |

**Table 2. Treatment Uptake Data Sources**

|  | **1995** | **Source** | **2008** | **Source** |
| --- | --- | --- | --- | --- |
| **MI** |  |  |  |  |
| Fibrinolysis | 15 | Assumed half of 2008 | 29 | Hospital based studies[[16](#_ENREF_16), [19](#_ENREF_19)] |
| Primary PCI | 4 | Assumed half of 2008 | 8 | Hospital based studies[[16](#_ENREF_16), [19](#_ENREF_19)] |
| Aspirin | 42 | Assumed half of 2008 | 93 | Hospital based studies[[16](#_ENREF_16), [19](#_ENREF_19)] |
| Beta Blockers | 35 | Assumed half of 2008 | 70 | Hospital based studies[[16](#_ENREF_16), [19](#_ENREF_19)] |
| ACE Inhibitor | 31 | Assumed half of 2008 | 62 | Hospital based studies[[16](#_ENREF_16), [19](#_ENREF_19)] |
| Primary CABG | 5 | Assumed half of 2008 | 9 | Hospital based studies[[16](#_ENREF_16), [19](#_ENREF_19)] |
| Clopidogrel |  |  |  | Hospital based studies[[16](#_ENREF_16), [19](#_ENREF_19)] |
| Community CPR |  |  |  |  |
| Hospital CPR | 3 | Assumed half of 2008 | 6 |  |
| Statin |  |  |  |  |
|  |  |  |  |  |
| **Angina** |  |  |  |  |
| Aspirin alone |  | Assumed half of 2008 | 50 | Hospital based studies[[16](#_ENREF_16), [19](#_ENREF_19)] [[40](#_ENREF_40)] |
| Aspirin and heparin |  | Assumed half of 2008 | 88 | Hospital based studies[[16](#_ENREF_16), [19](#_ENREF_19)] [[40](#_ENREF_40)] |
| Platelet glycoprotein IIB/IIIA inhibitors |  | Assumed half of 2008 | 41 | Hospital based studies[[16](#_ENREF_16), [19](#_ENREF_19)] [[40](#_ENREF_40)] |
| PCI |  |  | 13 | Hospital based studies[[16](#_ENREF_16), [19](#_ENREF_19)] [[40](#_ENREF_40)] |
| CABG surgery |  |  | 26 | Hospital based studies[[16](#_ENREF_16), [19](#_ENREF_19)] [[40](#_ENREF_40)] |
| Clopidogrel |  |  |  | Hospital based studies[[16](#_ENREF_16), [19](#_ENREF_19)] [[40](#_ENREF_40)] |
| **2^nd^ Prevention post AMI** |  |  |  |  |
| Aspirin | 39 | Assumed half of 2008 | 78 | Treatment uptake levels were estimated using results of hospital based studies[[16](#_ENREF_16), [19](#_ENREF_19)] [[40](#_ENREF_40)], EUROASPIRE-III[[17](#_ENREF_17)] and  PREMISE-II[[18](#_ENREF_18)] supplemented with expert opinion. |
| Beta Blokers | 27 |  | 53 |  |
| ACE inhibitors | 25 |  | 51 |  |
| Statins | 0 |  | 76 |  |
| Warfarin | 1.6 |  | 3.2 |  |
| Rehabilitation | 4.2 |  | 8.3 |  |
| **Chronic angina** |  |  |  |  |
| CABG surgery | 0 |  | 100 | EUROASPIRE-III[[17](#_ENREF_17)] supplemented with expert opinion. |
| Angioplasty | 0 |  | 100 | EUROASPIRE-III[[17](#_ENREF_17)] supplemented with expert opinion. |
| Aspirin | 20 | Assumed half of the last year | 44 | EUROASPIRE-III[[17](#_ENREF_17)] supplemented with expert opinion. |
| Statins | 0 |  | 20 | EUROASPIRE-III[[17](#_ENREF_17)] supplemented with expert opinion. |
| ACE inhibitors |  |  |  | EUROASPIRE-III[[17](#_ENREF_17)] supplemented with expert opinion. |
| **Hospital heart failure** |  |  |  |  |
| ACE inhibitors | 21 | 50% reduction of treatment uptake upon discharge obtained as expert opinion. | 42.5 | Hospital based treatment uptake studies[[16](#_ENREF_16), [19](#_ENREF_19), [41](#_ENREF_41)] supplemented with expert opinion. |
| Beta blockers | 18 | 50% reduction of treatment uptake upon discharge obtained as expert opinion. | 37.3 | Hospital based treatment uptake studies[[16](#_ENREF_16), [19](#_ENREF_19), [41](#_ENREF_41)] supplemented with expert opinion. |
| Spiroolactone | 20 | 50% reduction of treatment uptake upon discharge obtained as expert opinion. | 39.9 | Hospital based treatment uptake studies[[16](#_ENREF_16), [19](#_ENREF_19), [41](#_ENREF_41)] supplemented with expert opinion. |
| Aspirin | 34 | 50% reduction of treatment uptake upon discharge obtained as expert opinion. | 67.9 | Hospital based treatment uptake studies[[16](#_ENREF_16), [19](#_ENREF_19), [41](#_ENREF_41)] supplemented with expert opinion. |
| Statins |  |  | 0.000 |  |
| **Primary prevention hypertension** |  |  |  |  |
| Treated % | 25 | Assumed half of last year | 57.5 | Balçova Heart Study[[13](#_ENREF_13)] |
| **Primary prevention hyperlipidemia** |  |  |  |  |
| Treated % |  |  |  |  |
| Statins | 0 |  | 10.6 | Balçova Heart Study[[13](#_ENREF_13)] |

**Table 3. Age-Specific Case Fatality Rates for Each Patient Group**

|  | **AMI** | **POST AMI** | **Unstable angina** | **CABG** | **Angioplasty** | **HF in hospital** | **HF in community** | **Hypertension** | **Statins for primary prevention** |
| --- | --- | --- | --- | --- | --- | --- | --- | --- | --- |
| M 25-34 | 0.0110 | 0.0080 | 0.0160 | 0.0030 | 0.0030 | 0.0340 | 0.0110 | 0.0000 | 0.0000 |
| M 35-44 | 0.0120 | 0.0090 | 0.0240 | 0.0050 | 0.0050 | 0.0680 | 0.0220 | 0.0010 | 0.0010 |
| M 45-54 | 0.0230 | 0.0170 | 0.0340 | 0.0070 | 0.0070 | 0.0960 | 0.0320 | 0.0020 | 0.0020 |
| M 55-64 | 0.0540 | 0.0340 | 0.0560 | 0.0120 | 0.0120 | 0.1400 | 0.0450 | 0.0060 | 0.0060 |
| M 65-74 | 0.1010 | 0.0730 | 0.0700 | 0.0230 | 0.0250 | 0.2830 | 0.0930 | 0.0140 | 0.0140 |
| M 75+ | 0.1640 | 0.1220 | 0.0910 | 0.0420 | 0.0420 | 0.3370 | 0.1110 | 0.0350 | 0.0350 |
|  |  |  |  |  |  |  |  |  |  |
| F 25-34 | 0.0110 | 0.0040 | 0.0160 | 0.0030 | 0.0030 | 0.0340 | 0.0110 | 0.0000 | 0.0000 |
| F 35-44 | 0.0130 | 0.0060 | 0.0240 | 0.0050 | 0.0050 | 0.0680 | 0.0220 | 0.0010 | 0.0010 |
| F 45-54 | 0.0260 | 0.0100 | 0.0340 | 0.0070 | 0.0070 | 0.0960 | 0.0320 | 0.0010 | 0.0010 |
| F 55-64 | 0.0610 | 0.0190 | 0.0560 | 0.0120 | 0.0120 | 0.1400 | 0.0450 | 0.0020 | 0.0020 |
| F 65-74 | 0.1140 | 0.0840 | 0.0700 | 0.0230 | 0.0270 | 0.2220 | 0.0810 | 0.0070 | 0.0070 |
| F 75+ | 0.1670 | 0.1160 | 0.0910 | 0.0420 | 0.0390 | 0.2890 | 0.0940 | 0.0210 | 0.0210 |

# Table 4. Clinical efficacy of interventions: relative risk reductions obtained from meta-analyses, and randomised clinical trials

| **Treatments** | **Relative risk reduction^†^** | **Comments** | **Source paper: First author (year), notes** |
| --- | --- | --- | --- |
| ***ST elevation myocardial infarction (STEMI)*** | | | |
| **Thrombolysis** | 31% (95% CI: 14,45) | <55 years: Odds Ratio (OR)=0.692; Relative Risk Reduction (RRR)=30.8% (95% CI: 14,45)  55-64 years: OR=0.736; RRR=26.4% (95% CI: 17,40)  65-74 years: OR=0.752; RRR=24.8% (95% CI: 15,37)  > 75 years: OR=0.844; RRR=15.6% (95% CI: 4,30) | Estess (2002)[[42](#_ENREF_42)] |
| **Aspirin** | 23% (95% CI: 15,30) | RRR=23% (95% CI: 15,30): outcome is vascular death | ISIS-2 (1988)[[43](#_ENREF_43)] |
| **Primary CABG surgery** | 39% (95% CI: 23,52) | OR=0.61 (95% CI: 0.48,0.77); RRR=39% (95% CI: 23,52) on page 565, 0-5 year mortality | Yusuf (1994)[[44](#_ENREF_44)] |
| **Primary PCI** | 30% (95% CI: 15,42) | OR=0.70 (95% CI: 0.58,0.85); RRR=30% (95% CI: 15,42) outcome compares primary angioplasty to thrombolytics. | Keeley (2003)[[45](#_ENREF_45)] |
| **Beta blockers** | 4% (95% CI: -8,15) | OR=0.96 (95% CI: 0.85,1.08); RRR=4% (95% CI: -8,15) on page 1732 | Freemantle (1999)[[46](#_ENREF_46)] |
| **ACE inhibitors** | 7% (95% CI: 2,11) | OR=0.93 (95% CI: 0.89,0.98); RRR=7% (95% CI: 2,11) for 30 day mortality in myocardial infarction | ACE Inhibitor Myocardial Infarction Collaborative Group (1998)[[47](#_ENREF_47)] |
| **Clopidogrel** | 3% (95% CI: 1,6) | RRR=3% (95% CI: 1,6) for 30 day mortality in myocardial infarction | Chen (2005)[[48](#_ENREF_48)]  Sabatine (2005)[[49](#_ENREF_49)] |
| **Hospital CPR** | 33% (95% CI: 10,36) | Survival at 24 hours estimated to be 32%, discharge to home at 21%, and 1 year survival to be 15% overall. | Tunstall-Pedoe (1992)[[50](#_ENREF_50)]  Nadkarni[[51](#_ENREF_51)] |
| ***Non-ST-segment elevation acute coronary syndrome (NSTEACS):*** | | | |
| **Aspirin alone** | 15% (95% CI: 11,19) | OR=0.85 (95% CI: 0.49,0.95); RRR=15% (95% CI: 11,19). Outcome is vascular and nonvascular deaths on page 75. Assume appropriate for patients with NSTE-ACS. | Antithrombotic Trialists’ Collaboration (2002)[[20](#_ENREF_20)] |
| **Aspirin & heparin** | 33% (95% CI: -2,56) | OR=0.67 (95% CI: 0.48,1.02); RRR=33% (95% CI: -2,56%) in Table 2. The study outcome is composite MI death and non-fatal MI; compares those on aspirin & heparin to aspirin only. | Oler (1996)[[52](#_ENREF_52)] |
| **Platelet glycoprotein IIB/IIIA inhibitors** | 9% (95% CI: 2,16) | OR=0.91 (95% CI: 0.84,0.98); RRR=9% (95% CI: 2,16). Study looked at acute coronary syndrome without persistent ST elevation. | Boersma (2002)[[53](#_ENREF_53)] |
| **Early PCI** | 32% (95% CI: 5,51) | OR=0.68 (95% CI: 0.49,0.95); RRR=32% (95% CI: 5,51) | RITA 3 (Fox 2002)[[54](#_ENREF_54)] |
| **Primary CABG surgery** | 39% (95% CI: 23,52) | OR=0.61 (95% CI: 0.48,0.77); RRR=39% (95% CI: 23,52) on page 565, 0-5 year mortality | Yusuf (1994)[[44](#_ENREF_44)].  Assumed similar as STEMI. |
| **Clopidogrel** | 7% (95% CI: 2,11) | RRR=7% (95% CI: 2,11) | Yusuf (2001)[[55](#_ENREF_55)] |
| **Beta blockers** | 4% (95% CI: -8,15) | OR=0.96 (95% CI: 0.85,1.08); RRR=4% (95% CI: -8,15) on page 1732 | Freemantle (1999)[[46](#_ENREF_46)]  Assumed similar as STEMI. |
| **ACE inhibitors** | 7% (95% CI: 2,11) | OR=0.93 (95% CI: 0.89,0.98); RRR=7% (95% CI: 2,11) for 30 day mortality in myocardial infarction | ACE Inhibitor Myocardial Infarction Collaborative Group (1998)[[47](#_ENREF_47)] |
| ***Secondary prevention post myocardial infarction/revascularisation:*** | | | |
| **Aspirin** | 15% (95% CI: 11,19) | OR=0.85 (95% CI: 0.49,0.95); RRR=15% (95% CI: 11,19). Outcome is vascular and nonvascular deaths on page 75. This data seems to be appropriate to this outcome in CHD patients. | Antithrombotic Trialists’ Collaboration (2002)[[20](#_ENREF_20)] |
| **Beta blockers** | 23% (95% CI: 15,31) | OR=0.77 (95% CI: 0.69,0.85); RRR=23% (95% CI: 15,31) on page 1734. Odds of death in long term trials. | Freemantle (1999)[[46](#_ENREF_46)] |
| **ACE inhibitors or Angiotensin-II receptor antagonists** | 20% (95% CI: 13,26) | OR=0.80 (95% CI: 0.74,0.87); RRR=20% (95% CI: 13,26) on page 1577, death up to four years [endpoint of study looking at those with heart failure or LV dysfunction]. | Flather (2000)[[56](#_ENREF_56)] |
| **Statins** | 24% (95% CI: 10,26) | RRR=24% (95% CI: 10,26)  Intensive statin therapy in acute coronary syndromes. | Pignone (2006)[[57](#_ENREF_57)] |
| **Warfarin** | 22% (95% CI: 13,31) | OR=0.78 (95% CI: 0.67,0.90); RRR=22% (95% CI: 10,33) | Anand and Yusuf (1999)[[58](#_ENREF_58)] |
| **Rehabilitation** | 26% (95% CI: 10,39) | OR=0.74 (95% CI: 0.61,0.90); RRR=26% (95% CI: 10,39) in Figure 1, page 685 Taylor reference | Taylor (2004)[[59](#_ENREF_59)] |
| ***Chronic stable coronary artery disease:*** | | | |
| **CABG surgery**  **years 0-5** | 39% (95% CI:23,52) | OR = 0.61 (95% CI: 0.48-0.77), RRR 39% (95% CI: 23,52) on page 565, 5 year mortality | Yusuf (1994)[[44](#_ENREF_44)] |
| **CABG surgery**  **years 6-10** | 32% (95% CI: 2,30) | OR = 0.83 (95% CI: 0.70-0.98), RRR 17% (95% CI: 2,30) on page 565, 10 year mortality.  OR = 0.68 (95% CI: 0.56-0.83), RRR 32% (95% CI: 17,44) on page 565, 7 year mortality  CABG compared to medical treatment | Yusuf (1994)[[44](#_ENREF_44)] |
| **Angioplasty** | No effect |  | Boden (2007)[[60](#_ENREF_60)] |
| **Aspirin** | 15% (95% CI: 11,19) | OR=0.85 (95% CI: 0.49-0.95); RRR=15% (95% CI: 11,19). Outcome is vascular and nonvascular deaths on page 75. | Antithrombotic Trialists’ Collaboration (2002)[[20](#_ENREF_20)] |
| **Statins** | 23% (95% CI: 10,26) | RRR=23% (95% CI 10,26)  Standard dose statin therapy in coronary artery disease. | Wilt (2004)[[61](#_ENREF_61)] |
| **ACE inhibitors/ARB** | 17% (95% CI: 6,28) | RRR=17% (95% CI 6,28) | Al-Mallah (2006)[[62](#_ENREF_62)] |
| ***Heart failure in patients requiring hospitalisation or in the community:*** | | | |
| **ACE inhibitors** | 20% (95% CI: 13,26) | OR=0.80 (95% CI: 0.74,0.87); RRR=20% (95% CI: 13,26) on page 1577 [death up to four years was study endpoint for those with heart failure or LV dysfunction] | Flather (2000)[[56](#_ENREF_56)] |
| **Beta blockers** | 35% (95% CI: 26,43) | OR=0.65 (95% CI: 0.57,0.74); RRR=35% (95% CI: 26,43): all cause mortality | Shibata (2001)[[63](#_ENREF_63)] |
| **Spironolactone** | 30% (95% CI: 18,41)  31% (95% CI: 18,42) | OR=0.70 (95% CI: 0.59,0.82); RRR=30% (95% CI: 18,41) in those that had at least one cardiac related hospitalisation.  OR=0.69 (95% CI: 0.58,0.82); RRR=31% (95% CI: 18,42) in entire study population consisting of those with community heart failure, page 711. | Pitt (1999)[[64](#_ENREF_64)] |
| **Aspirin** | 15% (95% CI: 11,19) | OR=0.85 (95% CI: 0.49,0.95); RRR=15% (95% CI: 11,19). Outcome is vascular and nonvascular deaths on page 75. | Antithrombotic Trialists’ Collaboration (2002)[[20](#_ENREF_20)] |
| **Statins** | No effect |  | Kjekshus (2007)[[65](#_ENREF_65)]  Tavazzi (2008)[[66](#_ENREF_66)] |
| ***Primary prevention therapies:*** | | | |
| **Treatments for high blood pressure** | 13% (95% CI: 6,19) | OR=0.87 (95% CI: 0.81,0.94); RRR=13% (95% CI: 6,19) in those with high blood pressure without disease at entry. [RRR=29% (95% CI: 17,37) those with average blood pressure and CHD, treated with ACE inhibitors] | Law (2003)[[67](#_ENREF_67)] |
| **Statins** | 35% (95% CI: 11,52) | OR=0.65 (95% CI: 0.48,0.89); RRR=35% (95% CI: 11,52) for CHD mortality (only trials using statins), Figure 3 on page 4 | Pignone (2000)[[68](#_ENREF_68)] |

^†^Relative risk reduction (RRR) calculated as 1 – odds ratio

**eTable 5 Beta coefficients for major risk factors**

**Estimated β coefficients from multiple regression analyses for the relationship between absolute changes in population mean risk factors and percentage changes in coronary heart disease mortality for men and women, stratified by age. Data sources, values and comments.**

| **Systolic blood pressure** | | **Age group (years)** | | | | |
| --- | --- | --- | --- | --- | --- | --- |
|  | | **25-44** | **45-54** | **55-64** | **65-74** | **75+** |
| **Men** (hazard ratio per 20 mmHg) | | 0.49 | 0.49 | 0.52 | 0.58 | 0.65 |
| Men (log hazard ratio per 1 mmHg) | | **-0.036** | **-0.035** | **-0.032** | **-0.027** | **-0.021** |
| *Minimum* | | *-0.029* | *-0.028* | *-0.026* | *-0.022* | *-0.017* |
| *Maximum* | | *-0.043* | *-0.042* | *-0.039* | *-0.032* | *-0.025* |
|  | |  |  |  |  |  |
| **Women** (hazard ratio per 20 mmHg) | | 0.40 | 0.40 | 0.49 | 0.52 | 0.59 |
| Women (log hazard ratio per 1 mmHg) | | **-0.046** | **-0.046** | **-0.035** | **-0.032** | **-0.026** |
| *Minimum* | | *-0.037* | *-0.037* | *-0.028* | *-0.026* | *-0.021* |
| *Maximum* | | *-0.055* | *-0.055* | *-0.042* | *-0.039* | *-0.031* |
|  | |  |  |  |  |  |
| Source: Prospective studies collaborative meta-analysis, Lancet 2002[[69](#_ENREF_69)] | | | | | | |
| Units: Percentage change in CHD mortality per 20 mmHg change in systolic blood pressure | | | | | | |
| **Strengths:** | Large dataset, includes US data, adjusted for regression dilution bias, consistent with randomised controlled trials, results stratified by age and sex, with 95% confidence intervals | | | | | |
| **Limitations:** | Some publication bias still possible | | | | | |

| **Cholesterol** | **Age groups (years)** | | | | | | |
| --- | --- | --- | --- | --- | --- | --- | --- |
|  | **25-44** | | **45-54** | **55-64** | **65-74** | **75-84** | **85+** |
| **Mortality reduction per 1 mmol/l** | | | | | | | |
| Men | 0.55 | | 0.53 | 0.36 | 0.21 | 0.21 | 0.21 |
| Women | 0.57 | | 0.52 | 0.35 | 0.23 | 0.23 | 0.23 |
| **Log coefficient** | | | | | | | |
| **Men** | **-0.799** | | **-0.755** | **-0.446** | **-0.236** | **-0.117** | **-0.083** |
| *Minimum* | *-0.639* | | *-0.604* | *-0.357* | *-0.189* | *-0.093* | *-0.067* |
| *Maximum* | *-0.958* | | *-0.906* | *-0.536* | *-0.283* | *-0.140* | *-0.100* |
|  |  | |  |  |  |  |  |
| **Women** | **-0.844** | | **-0.734** | **-0.431** | **-0.261** | **-0.174** | **-0.051** |
| *Minimum* | *-0.675* | | *-0.587* | *-0.345* | *-0.209* | *-0.139* | *-0.041* |
| *Maximum* | *-1.013* | | *-0.881* | *-0.517* | *-0.314* | *-0.209* | *-0.062* |
| Source: Prospective studies collaborative meta-analysis, Lancet 2007[[70](#_ENREF_70)] | | | | | | | |
| Units: | | Percentage change in CHD mortality per 1 mmol/l change in total cholesterol | | | | | |
| **Strengths:** | | Includes US data, adjusted for regression dilution bias, includes randomised controlled trials, RCT values consistent with observational data, results stratified by age and sex, with 95% confidence intervals | | | | | |
| **Limitations:** | | Some publication bias still possible | | | | | |

| **Body Mass Index (BMI)** | | **Age groups (years)** | | | | |
| --- | --- | --- | --- | --- | --- | --- |
|  | | **<44** | **45-59** | **60-69** | **70-79** | **80+** |
| *James et.al (2004):* | |  |  |  |  |  |
| Hazard ratio | | 0.89 | 0.91 | 0.95 | 0.96 | 0.97 |
| Risk reduction† per 1 kg/m^2^ | | 0.11 | 0.09 | 0.05 | 0.04 | 0.03 |
| Age gradient (45-59 as reference) | | 1.22 | **1.00** | 0.56 | 0.44 | 0.33 |
| *Bogers (2006):*  Relative risks, CHD deaths per 5 BMI units (kg/m^2^) | |  | **1.16** |  |  |  |
| Relative risks per 1 kg/m^2^ applying age gradients from James et.al | | 1.04 | 1.03 | 1.02 | 1.01 | 1.01 |
| **Log coefficients** | | **0.0363** | **0.0297** | **0.0165** | **0.0132** | **0.0099** |
| *Minimum* | | *0.0255* | *0.0209* | *0.0116* | *0.0093* | *0.0070* |
| *Maximum* | | *0.0466* | *0.0381* | *0.0212* | *0.0169* | *0.0127* |
| Source: Bogers et al (2006)[[71](#_ENREF_71)], James et al (2004)[[72](#_ENREF_72)] | | | | | | |
| Units: | Percentage change in CHD mortality per 1 kg/m^2^ change in BMI | | | | | |
| **Strengths:** | Large number of studies included. Adjusted for blood pressure, total cholesterol, and physical activity. 95% confidence intervals included. | | | | | |
| **Limitations:** | Observational data; age gradient applied from James study | | | | | |

^†^ Risk reduction = 1 – hazard ratio

**References**

1. Capewell S, Morrison CE, McMurray JJ: **Contribution of modern cardiovascular treatment and risk factor changes to the decline in coronary heart disease mortality in Scotland between 1975 and 1994**. *Heart* 1999, **81**(4):380-386.

2. Capewell S, Beaglehole R, Seddon M, McMurray J: **Explaining the decline in Coronary Heart Disease Mortality in Auckland, New Zealand between 1982 and 1993**. *Circulation* 2000, **102**:1511-1516.

3. Unal B, Critchley JA, Capewell S: **Explaining the decline in coronary heart disease mortality in England and Wales between 1981 and 2000**. *Circulation* 2004, **109**(9):1101-1107.

4. Critchley J, Liu J, Zhao D, Wei W, Capewell S: **Explaining the increase in coronary heart disease mortality in Beijing between 1984 and 1999**. *Circulation* 2004, **110** 1236-1244

5. Ford ES, Ajani UA, Croft JB, Critchley JA, Labarthe DR, Kottke TE, Giles WH, Capewell S: **Explaining the decrease in U.S. deaths from coronary disease, 1980-2000**. *N Engl J Med* 2007, **356**(23):2388-2398.

6. *Population statistics*. Ankara: Turkish Statistical Institute (TUIK); 2011.

7. **Mortality statistics 1988-2008** [<http://tuikapp.tuik.gov.tr/demografiapp/olum.zul>]

8. *National burden of disease and cost effectiveness studies reports*. Ankara: Ministry of Health, Turkey; 2007.

9. **Appendices for IMPACT CHD Mortality Model** [<http://www.liv.ac.uk/PublicHealth/sc/bua/IMPACT_Model_%20Appendices_May_2007.pdf>]

10. **Health Survey 2008**. Ankara: Turkish Statistical Institute; 2009.

11. Degertekin M, Erol C, Ergene O, Tokgozoglu L, Aksoy M, Erol MK, Eren M, Sahin M, Eroglu E, Mutlu B *et al*: **[Heart failure prevalence and predictors in Turkey: HAPPY study]**. *Turk Kardiyol Dern Ars* 2012, **40**(4):298-308.

12. Ergor G, Soysal A, Sozmen K, Unal B, Ucku R, Kilic B, Gunay T, Ergor A, Demiral Y, Saatli G *et al*: **Balcova heart study: rationale and methodology of the Turkish cohort**. *Int J Public Health* 2011.

13. Unal B, Sozmen K, Ucku R, Ergor G, Soysal A, Baydur H, Meseri R, Simsek H, Gerceklioglu G, Doganay S *et al*: **High prevalence of cardiovascular risk factors in a Western urban Turkish population: a community-based study**. *Anadolu Kardiyol Derg* 2012.

14. Badillioglu O UB, Ucku R: **Five-year incidence of coronary heart disease and risk factors in Güzelbahçe, İzmir**. *Turkish Journal of Public Health* 2011, **9**(3).

15. Onat A, Can G, Hergenc G, Kucukdurmaz Z, Ugur M, Yuksel H: **High absolute coronary disease risk among Turks: involvement of risk factors additional to conventional ones**. *Cardiology* 2010, **115**(4):297-306.

16. Aslan BU, Karcioglu O, Aslan O, Ayrik C, Kulac E, Guneri S: **[Does the short-term mortality differ between men and women with first acute myocardial infarction?]**. *Anadolu Kardiyol Derg* 2002, **2**(4):284-290.

17. Tokgözoğlu L KE, Erol C, Ergene O; EUROASPIRE III Turkey Study Group.: **[EUROASPIRE III: a comparison between Turkey and Europe].** *Turk Kardiyol Dern Ars* 2010, **38**(3):164-172

18. Mendis S, Abegunde D, Yusuf S, Ebrahim S, Shaper G, Ghannem H, Shengelia B: **WHO study on Prevention of REcurrences of Myocardial Infarction and StrokE (WHO-PREMISE)**. *Bull World Health Organ* 2005, **83**(11):820-829.

19. Simsek H, Demiral Y, Aslan O, Unal B: **Treatment uptake levels in coronary heart disease patients at hospital discharge**. *International Journal of Cardiology* 2011, **147**:S156-S156.

20. **Collaborative meta-analysis of randomised trials of antiplatelet therapy for prevention of death, myocardial infarction, and stroke in high risk patients**. *BMJ* 2002, **324**(7329):71-86.

21. Capewell S, Livingston BM, MacIntyre K, et al.: **Trends in case-fatality in 117 718 patients admitted with acute myocardial infarction in Scotland**. *EurHeart J* 2000, **21**(22):1833-1840.

22. Lewington S, Clarke R, Qizilbash N, Peto R, Collins R: **Age-specific relevance of usual blood pressure to vascular mortality: a meta-analysis of individual data for one million adults in 61 prospective studies**. *Lancet* 2002, **360**(9349):1903-1913.

23. Yusuf S, Hawken S, Ounpuu S, Dans T, Avezum A, Lanas F, McQueen M, Budaj A, Pais P, Varigos J *et al*: **Effect of potentially modifiable risk factors associated with myocardial infarction in 52 countries (the INTERHEART study): case-control study**. *Lancet* 2004, **364**(9438):937-952.

24. Mant J, Hicks N: **Detecting differences in quality of care: the sensitivity of measures of process and outcome in treating acute myocardial infarction**. *BMJ* 1995, **311**(7008):793-796.

25. Yusuf S, Pais P, Afzal R, Xavier D, Teo K, Eikelboom J, Sigamani A, Mohan V, Gupta R, Thomas N: **Effects of a polypill (Polycap) on risk factors in middle-aged individuals without cardiovascular disease (TIPS): a phase II, double-blind, randomised trial**. *Lancet* 2009, **373**(9672):1341-1351.

26. Wald NJ, Law MR: **A strategy to reduce cardiovascular disease by more than 80%**. *BMJ* 2003, **326**(7404):1419.

27. Briggs A, Sculpher M, Buxton M: **Uncertainty in the economic evaluation of health care technologies: the role of sensitivity analysis**. *Health Economics* 1994, **3**:95-104.

28. **Hospital admission statistics. Distribution of inpatients by 150 selected diseasesl in Turkey** Turkey Statistical Institute; 2012.

29. Wilson PW, D'Agostino RB, Levy D, Belanger AM, Silbershatz H, Kannel WB: **Prediction of coronary heart disease using risk factor categories**. *Circulation* 1998, **97**(18):1837-1847.

30. **Health Survey 2010**. Ankara: Turkish Statistical Institute; 2010.

31. Altun B, Arici M, Nergizoglu G, Derici U, Karatan O, Turgan C, Sindel S, Erbay B, Hasanoglu E, Caglar S: **Prevalence, awareness, treatment and control of hypertension in Turkey (the PatenT study) in 2003**. *J Hypertens* 2005, **23**(10):1817-1823.

32. Erem C, Hacihasanoglu A, Deger O, Kocak M, Topbas M: **Prevalence of dyslipidemia and associated risk factors among Turkish adults: Trabzon lipid study**. *Endocrine* 2008, **34**(1-3):36-51.

33. Satman I: **TURDEP II: Initial Findings**. 2010.

34. Sanisoglu SY, Oktenli C, Hasimi A, Yokusoglu M, Ugurlu M: **Prevalence of metabolic syndrome-related disorders in a large adult population in Turkey**. *BMC Public Health* 2006, **6**:92.

35. Satman I, Yilmaz T, Sengul A, Salman S, Salman F, Uygur S, Bastar I, Tutuncu Y, Sargin M, Dinccag N *et al*: **Population-based study of diabetes and risk characteristics in Turkey: results of the turkish diabetes epidemiology study (TURDEP)**. *Diabetes Care* 2002, **25**(9):1551-1556.

36. *Global Adult Tobacco Survey Turkey Report*. Ankara: The Ministry of Health of Turkey; 2010.

37. Tezcan S, Altintas H, Sonmez R, Akinci A, Dogan B, Cakir B, Bilgin Y, Klor HU, Razum O: **Cardiovascular risk factor levels in a lower middle-class community in Ankara, Turkey**. *TropMedIntHealth* 2003, **8**(7):660-667.

38. Onat A: **Lipids, lipoproteins and apolipoproteins among turks, and impact on coronary heart disease**. *AnadoluKardiyolDerg* 2004, **4**(3):236-245.

39. Onat A: **Risk factors and cardiovascular disease in Turkey**. *Atherosclerosis* 2001, **156**(1):1-10.

40. Sonmez K, Akcay A, Akcakoyun M, Demir D, Elonu OH, Onat O, Duran NE, Gencbay M, Degertekin M, Turan F: **[Distribution of therapeutic procedures and choice of drug therapies in patients with angiographically confirmed coronary artery disease]**. *AnadoluKardiyolDerg* 2002, **2**(1):18-23, AXVI.

41. Ergin A EN, Ünal Ş, Delice A, Topsakal R, Seyfeli E. : **Epidemiological and Pharmacological Profile of Congestive Heart Failure at Turkish Academic Hospitals.** *Anatolian Journal of Cardiology* 2004, **4**:32-38.

42. Estess JM, Topol EJ: **Fibrinolytic treatment for elderly patients with acute myocardial infarction**. *Heart* 2002, **87**(4):308-311.

43. Baigent C, Collins R, Appleby P, Parish S, Sleight P, Peto R: **ISIS-2: 10 year survival among patients with suspected acute myocardial infarction in randomised comparison of intravenous streptokinase, oral aspirin, both, or neither. The ISIS-2 (Second International Study of Infarct Survival) Collaborative Group**. *BMJ* 1998, **316**(7141):1337-1343.

44. Yusuf S, Zucker D, Peduzzi P, Fisher LD, Takaro T, Kennedy JW, Davis K, Killip T, Passamani E, Norris R: **Effect of coronary artery bypass graft surgery on survival: overview of 10-year results from randomised trials by the Coronary Artery Bypass Graft Surgery Trialists Collaboration**. *Lancet* 1994, **344**(8922):563-570.

45. Keeley EC, Velez CA, O'Neill WW, Safian RD: **Long-term clinical outcome and predictors of major adverse cardiac events after percutaneous interventions on saphenous vein grafts**. *JAmCollCardiol* 2001, **38**(3):659-665.

46. Freemantle N, Cleland J, Young P, Mason J, Harrison J: **beta Blockade after myocardial infarction: systematic review and meta regression analysis**. *BMJ* 1999, **318**(7200):1730-1737.

47. **Indications for ACE inhibitors in the early treatment of acute myocardial infarction: systematic overview of individual data from 100,000 patients in randomized trials. ACE Inhibitor Myocardial Infarction Collaborative Group**. *Circulation* 1998, **97**(22):2202-2212.

48. Chen ZM, Jiang LX, Chen YP, Xie JX, Pan HC, Peto R, Collins R, Liu LS: **Addition of clopidogrel to aspirin in 45,852 patients with acute myocardial infarction: randomised placebo-controlled trial**. *Lancet* 2005, **366**(9497):1607-1621.

49. Sabatine MS, Cannon CP, Gibson CM, Lopez-Sendon JL, Montalescot G, Theroux P, Claeys MJ, Cools F, Hill KA, Skene AM *et al*: **Addition of clopidogrel to aspirin and fibrinolytic therapy for myocardial infarction with ST-segment elevation**. *N Engl J Med* 2005, **352**(12):1179-1189.

50. Tunstall-Pedoe H, Bailey L, Chamberlain DA, et al.: **Survey of 3765 cardiopulmonary resuscitations in British hospitals (the BRESUS Study): methods and overall results**. *BMJ* 1992, **304**(6838):1347-1351.

51. Nadkarni VM, Nolan JP, Billi JE, Bossaert L, Bottiger BW, Chamberlain D, Drajer S, Eigel B, Hazinski MF, Hickey RW *et al*: **Part 2: International collaboration in resuscitation science: 2010 International Consensus on Cardiopulmonary Resuscitation and Emergency Cardiovascular Care Science With Treatment Recommendations**. *Circulation* 2010, **122**(16 Suppl 2):S276-282.

52. Oler A, Whooley MA, Oler J, Grady D: **Adding heparin to aspirin reduces the incidence of myocardial infarction and death in patients with unstable angina. A meta-analysis**. *JAMA* 1996, **276**(10):811-815.

53. Boersma E, Harrington RA, Moliterno DJ, White H, Theroux P, Van de WF, de Torbal A, Armstrong PW, Wallentin LC, Wilcox RG *et al*: **Platelet glycoprotein IIb/IIIa inhibitors in acute coronary syndromes: a meta-analysis of all major randomised clinical trials**. *Lancet* 2002, **359**(9302):189-198.

54. Fox KA, Poole-Wilson PA, Henderson RA, Clayton TC, Chamberlain DA, Shaw TR, Wheatley DJ, Pocock SJ: **Interventional versus conservative treatment for patients with unstable angina or non-ST-elevation myocardial infarction: the British Heart Foundation RITA 3 randomised trial. Randomized Intervention Trial of unstable Angina**. *Lancet* 2002, **360**(9335):743-751.

55. Yusuf S, Zhao F, Mehta SR, Chrolavicius S, Tognoni G, Fox KK: **Effects of clopidogrel in addition to aspirin in patients with acute coronary syndromes without ST-segment elevation**. *N Engl J Med* 2001, **345**(7):494-502.

56. Flather MD, Yusuf S, Kober L, Pfeffer M, Hall A, Murray G, Torp-Pedersen C, Ball S, Pogue J, Moye L *et al*: **Long-term ACE-inhibitor therapy in patients with heart failure or left- ventricular dysfunction: a systematic overview of data from individual patients. ACE-Inhibitor Myocardial Infarction Collaborative Group**. *Lancet* 2000, **355**(9215):1575-1581.

57. Pignone M, Earnshaw S, Tice JA, Pletcher MJ: **Aspirin, statins, or both drugs for the primary prevention of coronary heart disease events in men: a cost–utility analysis**. *Annals of Internal Medicine* 2006, **144**(5):326.

58. Anand SS, Yusuf S: **Oral anticoagulant therapy in patients with coronary artery disease: a meta-analysis**. *JAMA* 1999, **282**(21):2058-2067.

59. Taylor RS, Brown A, Ebrahim S, Jolliffe J, Noorani H, Rees K, Skidmore B, Stone JA, Thompson DR, Oldridge N: **Exercise-based rehabilitation for patients with coronary heart disease: systematic review and meta-analysis of randomized controlled trials**. *Am J Med* 2004, **116**(10):682-692.

60. Boden WE, O'Rourke RA, Teo KK, Hartigan PM, Maron DJ, Kostuk WJ, Knudtson M, Dada M, Casperson P, Harris CL *et al*: **Optimal Medical Therapy with or without PCI for Stable Coronary Disease**. *New England Journal of Medicine* 2007, **356**(15):1503-1516.

61. Wilt TJ, Bloomfield HE, MacDonald R, et al.: **Effectiveness of statin therapy in adults with coronary heart disease**. *Archives of Internal Medicine* 2004, **164**(13):1427-1436.

62. Al-Mallah MH, Tleyjeh IM, Abdel-Latif AA, Weaver WD: **Angiotensin-converting enzyme inhibitors in coronary artery disease and preserved left ventricular systolic function: a systematic review and meta-analysis of randomized controlled trials**. *J Am Coll Cardiol* 2006, **47**(8):1576-1583.

63. Shibata MC, Flather MD, Wang D: **Systematic review of the impact of beta blockers on mortality and hospital admissions in heart failure**. *EurJHeart Fail* 2001, **3**(3):351-357.

64. Pitt B, Zannad F, Remme WJ, Cody R, Castaigne A, Perez A, Palensky J, Wittes J: **The Effect of Spironolactone on Morbidity and Mortality in Patients with Severe Heart Failure**. *New England Journal of Medicine* 1999, **341**(10):709-717.

65. Kjekshus J, Pedersen TR, Olsson AG, Faergeman O, Pyorala K: **The effects of simvastatin on the incidence of heart failure in patients with coronary heart disease**. *J Card Fail* 1997, **3**(4):249-254.

66. Tavazzi L, Maggioni AP, Marchioli R, Barlera S, Franzosi MG, Latini R, Lucci D, Nicolosi GL, Porcu M, Tognoni G: **Effect of rosuvastatin in patients with chronic heart failure (the GISSI-HF trial): a randomised, double-blind, placebo-controlled trial**. *Lancet* 2008, **372**(9645):1231-1239.

67. Law MR, Wald NJ, Morris JK, Jordan RE: **Value of low dose combination treatment with blood pressure lowering drugs: analysis of 354 randomised trials**. *BMJ* 2003, **326**(7404):1427.

68. Pignone M, Phillips C, Mulrow C: **Use of lipid lowering drugs for primary prevention of coronary heart disease: meta-analysis of randomised trials**. *BMJ* 2000, **321**(7267):983-986.

69. Lewington S, Clarke R, Qizilbash N, Peto R, Collins R: **Prospective Studies Collaboration. Age-specific relevance of usual blood pressure to vascular mortality: a meta-analysis of individual data for one million adults in 61 prospective studies**. *Lancet* 2002, **360**(9349):1903-1913.

70. Lewington S, Whitlock G, Clarke R, Sherliker P, Emberson J, Halsey J, Qizilbash N, Peto R, Collins R: **Prospective Studies Collaboration. Blood cholesterol and vascular mortality by age, sex, and blood pressure: a meta-analysis of individual data from 61 prospective studies with 55,000 vascular deaths**. *Lancet* 2007, **370**(9602):1829-1839.

71. Bogers RP, Bemelmans WJ, Hoogenveen RT, Boshuizen HC, Woodward M, Knekt P, van Dam RM, Hu FB, Visscher TL, Menotti A *et al*: **Association of overweight with increased risk of coronary heart disease partly independent of blood pressure and cholesterol levels: a meta-analysis of 21 cohort studies including more than 300 000 persons**. *Arch Intern Med* 2007, **167**(16):1720-1728.

72. James PT, Rigby N, Leach R, Force IOT: **The obesity epidemic, metabolic syndrome and future prevention strategies.** *Eur J Cardiovasc Prev Rehabil* 2004, **11**(1):3-8.
